# Proceedings of the 93rd Annual Business Meeting of the American Association of Biological Anthropologists

**DOI:** 10.1002/ajpa.70151

**Published:** 2025-11-13

**Authors:** Amy L. Rector

**Affiliations:** ^1^ Anthropology, School of World Studies Virginia Commonwealth University Richmond Virginia USA

President Leslea Hlusko called the meeting to order in Los Angeles, CA, at 6:30 p.m. Pacific Time on March 22, 2024. A quorum of over 40 Regular (voting) members present was established by the Secretary. The President welcomed attendees and read a land acknowledgment. Minutes of the 2023 Business Meeting were approved as presented. All Annual Reports were uploaded on the AABA website prior to the Business Meeting and members were encouraged to review them online. The President, Vice President, Treasurer, *American Journal of Biological Anthropology* and *Yearbook of Biological Anthropology* reports were also presented orally at the meeting.

## 1 Report of the President

Leslea Hlusko presented comments and provided the following written report:

*This report was jointly written by Past‐President Steve Leigh and President Leslea Hlusko. From July 1, 2023, until February 1, 2024, Leslea Hlusko was on medical leave and could not serve effectively as President of AABA. As per AABA by‐laws, the Past‐ President stepped in as Acting President. Consequently, most of the past years of AABA activities were under Steve Leigh's leadership through his role as Acting President*.

Please accept our appreciation and thanks to you, our members, for your involvement and dedication to the Association. As usual, during our annual business meeting, we'll engage in the routine business of our association and celebrate the accomplishments of our colleagues with awards. The business meeting will be broadcast live and recorded for those who are participating virtually this year.

The AABA has pursued numerous initiatives this year to address issues specific to the association as well as more general issues. Several initiatives have been substantial in their scope, involving tremendous commitment and hard work from many members. We thank all for their efforts and dedication to the discipline and to the AABA.

### 1.1 Annual Conference

The 2023 conference in Reno was well‐received by attendees, and while we had fewer presentations and registrations than recent pre‐pandemic conferences, our time in Reno was productive and engaging.

This year, registration fees were increased over 2023 levels, partly because of relatively higher anticipated expenses in Los Angeles (e.g., early registration increased from $215 to $230 for regular members, $176 to $190 for postdoc/contingent members, from $88 to $90 for student members), and there was no increase in registration cost for members who are retired ($90) or from any registering from a Qualifying or Tribal Nation ($25–$50). We have continued to work to minimize conference costs and registration fees. Registration fees were held at 2019 levels through 2022, despite some inflationary cost pressures, followed by a modest increase in 2023.

It should be noted that the Association subsidizes conference costs through investment income with the goal of keeping our member costs as low as possible. In fact, our registration fees remain remarkably low relative to peer organizations. Post‐pandemic meetings have had reduced attendance, highlighting scaling effects with our conferences. Specifically, high attendance results in lower marginal costs, better overall revenue and thus both less risk and cost pressure for the Association. Given that the three post‐pandemic meetings have had about 80% of the attendance of pre‐2020 conferences, we may need to plan for smaller conferences in the future. This will be discussed during the 2024 Presidential Panel in Los Angeles.

Our Vice President and Program Chair, Dr. Kristi Lewton skillfully organized our 2024 conference, beginning with receipt of symposium proposals in July. She has continued to innovate and update our annual conference. This year offers many new features that attendees will appreciate. It is with deep gratitude that we thank her for her incredible service to the Association. Along with Dr. Lewton, we thank our colleagues on the Program Committee for their work in several areas, including abstract review and composing scientific sessions. Our Local Arrangements Committee, Drs. Kristi Lewton, Stephanie Merdith, and David Raichlen collaborated with our Advance Team to plan the conference. Additional Advance Team members included Dr. Amy Rector, Dr. Eduardo Amorim, and, from Burke and Associates, Lori Strong. The team was assisted by student volunteers Katie Beachem, Chloe Coulter, Cassie Croasmun, Alexandria Koedel, and Anamika Nanda.

### 1.2 Concerns Regarding Labor Actions

Hotel workers' union actions complicated planning for this year's conference. Beginning in June, union workers engaged in actions against hotels, including our conference hotel. The Local Arrangements Committee carefully monitored the situation, and we issued a statement in support of the union and called for a resolution. During our Advance Team visit, we provided the hotel with a letter emphasizing aspects of our contract that would apply to this situation and calling for a resolution. Fortunately, the issue was settled in late 2023 and early 2024.

### 1.3 Meeting Sites for 2027–2029

The Executive Committee selected conference venues for 2027–2029 in December. The committee received information from Burk and Associates regarding 23 venues in numerous cities. The committee decided to select venues in states that have not passed legislation intended to limit individual and intellectual freedom along lines of gender, race, class, or other dimensions.

After coordination between Lori Strong from BAI, and following legal review of contracts, we signed the hotel contracts for AABA's conferences in 2027, 2028, and 2029:

○ Philadelphia Marriott Downtown, March 31–April 3, 2027
○ Chicago Marriott Downtown Magnificent Mile, April 19–22, 2028
○ San Francisco Marriott Marquis, April 4–7, 2029

### 1.4 Cognate Organization Cost Shares

Our annual conference involves logistical and other kinds of support for related organizations, including the American Association of Anthropological Genetics (AAAG), Dental Anthropology Association (DAA), Human Biology Association (HBA), Paleopathology Association (PPA) and, in alternating years, the Paleoanthropology Society (PAS). Of these groups, the HBA, PPA, and PAS offer programs supported by registration fees. Close evaluation of AABA conference budgets indicated that the AABA subsidizes elements of these programs. Therefore, the AABA initiated discussions with leadership of the HBA, PPA, and PAS regarding program costs and strategies to offset the subsidies from AABA registrations that support these programs. Discussions among the associations are ongoing and may involve future changes to registrations, including “bundling” registrations for AABA and related programs.

### 1.5 Harassment‐Avoidance Training

Starting in 2021, AABA began contracting with an outside firm to provide anti‐ harassment and active bystander training to conference session chairs and members of the Executive Committee. This was initially contracted with Sherry Marts at S*Marts Consulting. Paula Brantner provided this training for the 2023 conference. However, for 2024, AABA needed another option due to contract requirements with Paula Brantner that would have included services AABA does not need. For 2024, Vice President Kristi Lewton identified ADVANCEGeo (https://serc.carleton.edu/advancegeo/index.html), a multi‐institutional partnership funded in part by the National Science Foundation. This organization was hired to provide a 2.5 h workshop for AABA session chairs and Executive Committee on March 5, 2024.

### 1.6 Expansion of Qualifying Nations

Early Career Liaison Justin Lund initiated a proposal in collaboration with the Committee on Diversity and Student Programs Chair Chelsea Juarez to expand “Qualifying Nations” registration fees to include members of Native American Tribal Nations. The AABA has, for many years, discounted registration fees based on a World Bank classification of Middle‐ and Low‐income countries. The new registration system applies these fees to Tribal Members as well.

### 1.7 Changes to *In Memoriam*

Over the years, the number of AABA community members who pass away each year has reached the point where the Executive Committee felt like we could no longer adequately pay our respects given the time limitations of the annual Business Meeting. Therefore, for 2024, we are implementing a new approach to this important part of our annual meeting. The names of colleagues who have passed since our last annual meeting will be listed during the Business Meeting. Everyone is then invited to attend a poster session on Saturday afternoon, where each lost colleague will have a poster that provides an overview of their professional contributions.

### 1.8 AABA Award Winners

We are, once again, recognizing a remarkable group of scholars and colleagues who continue to make important contributions to our discipline. This year, Dr. Laurie R. Godfrey received the Charles R. Darwin Lifetime Achievement Award. Our Gabriel Lasker Service Distinguished Service Award goes to Dr. Steven R. Leigh. Dr. Tina Lasisi received the AABA and Leakey Foundation Communication and Outreach Award in Honor of Camilla M. Smith. We also extend a hearty congratulations to this year's Cobb Professional Development Awards and the Student Presentation Prize and Pollitzer Travel Award winners.

### 1.9 Constitution and By‐Laws Amendment

In 2022, a proposal was submitted in accordance with our *Constitution and By‐Laws* to change the AABA *Constitution and By‐Laws* to align the roles and responsibilities of Local Arrangements Committees with current practices. More specifically, the document stipulates major responsibilities for the Local Arrangements Committee, including contractual and financial responsibilities, along with logistics, accounting and other tasks. These responsibilities are now handled by Burk and Associates in close collaboration with Association officers (President, Vice President, Treasurer, and Secretary).

During the Annual Business Meeting in 2023, Regular Members approved an amendment to the text of the AABA *Constitution and By‐Laws* regarding Article III *Committees*, *Section 2b, Ad Hoc Committees*.

The current text states:

*Section 2b. Ad Hoc Committees*. Each annual meeting of the Association shall be managed by a Local Arrangements Chair appointed by the President, with approval of the Executive Committee, to serve for a term determined by the President. The Local Arrangements Chair is responsible for planning the meeting, overseeing and managing its operations and finances and bringing them to a conclusion, giving a final accounting, and reporting on these activities to the President, the Executive Committee, and the business meeting of the Association. Each Local Arrangements Chair is empowered to appoint an *ad hoc* Local Arrangements Committee to assist in carrying out these duties. Since each meeting will ordinarily have its own Local Arrangements Chair, there may be several such Chairs serving concurrently. The President may, when necessary and with the consent of a majority of the members of the Executive Committee, appoint other *ad hoc* committees to deal with specific issues. *Ad hoc* committees exist for a period of up to three years. Additional years may be added by the President and Executive Committee.

The proposal to amend this text was approved via voice vote by Regular Members during the Annual Business Meeting, with a member request to consider a slight change to the wording of the proposed text. The Executive Committee unanimously approved the request for a minor change to the wording of the document, shown in italics:

From: “The Local Arrangements Committee is responsible for *supporting* the Vice President and Program Chair *with* local matters related to the annual meetings.”

To: “The Local Arrangements Committee is responsible for *collaborating with* the Vice President and Program Chair *on* local matters related to the annual meetings.”

The proposed text offered for a vote during the 2024 Annual Business Meeting states:

*Section 2b. Ad Hoc Committees*. Each annual meeting of the Association shall be supported by a Local Arrangements Committee appointed by the President to serve until the conclusion of the annual meeting. The Local Arrangements Committee is responsible for collaborating with the Vice President and Program Chair on local matters related to the annual meetings. The President may, when necessary and with the consent of a majority of the members of the Executive Committee, appoint other *ad hoc* committees to deal with specific issues. *Ad hoc* committees exist for a period of up to three years. Additional years may be added by the President and Executive Committee.

An affirmative vote will result in this text being incorporated into the *Constitution and By‐Laws*.

### 1.10 Executive Committee Activities

The Executive Committee continued its work on behalf of the Association and its members. This included routine tasks, such as award selections, committee appointments, website and data infrastructure updates, and conference planning. Beyond standard duties and responsibilities, the committee was involved in developing a number of public statements this year, covering a wide range of issues (see Section 1.18). In addition, the group undertook deliberations on future conference venues, evaluated cost share proposals (see Section 1.4), and made decisions regarding web and conference resources. All of this work will benefit the Association well into the future.

### 1.11 Elected Membership Changes

The 2024 annual conference brings substantial changes to the membership of our Executive Committee, with members rotating out of positions and newly elected colleagues joining the group. This year, five members will either be departing leadership roles or moving to new roles. All of these colleagues have served the association with distinction. Past and Acting President Steve Leigh will be leaving the committee after serving consecutively as Vice President and Program Chair, President Elect, President, and Past and Acting President (since 2018). Dr. Leigh is being recognized at the annual Business Meeting with the Gabriel W. Lasker Service Award for his incredible dedication to the Association over the last 6 years, leading AABA through a number of unprecedented situations with immense grace and adeptness.

Vice President and Program Chair, Dr. Kristi Lewton will be taking on a role as Secretary. Dr. Lewton produced our 2023 and 2024 meetings with careful attention to scientific rigor and with a keen eye on the highest scientific standards. In addition, Dr. Lewton has been deeply involved in restructuring our web presence and in moving towards a new electronic conference platform, preparing the Association well for the future.

Dr. Amy Rector completes her term as Secretary and will go on to serve as our new Vice President and Program Chair. Dr. Rector has made significant contributions to the association through committee work on the web and conference system committee. She has meticulously conducted her work as Secretary while aiding the Association with social media and communication efforts.

History and Honors Chair, Dr. Julienne Rutherford has worked diligently and with immense attention to transparency to support our colleagues who have been nominated for awards as well as those who have won these awards. In addition, she led efforts to preserve and celebrate the history of our discipline.

Dr. Chelsey Juarez, Student Programs Chair, has worked tirelessly on behalf of our student members. She and her committee have worked to expand opportunities for all students, including evaluation of Pollitzer Travel Awards and conferral of student awards. Her efforts have helped our remarkable students begin their careers as professionals in our field.

We thank these wonderful colleagues who have worked so hard for the benefit of the Association, and hope that they continue their involvement with the AABA in the future.

Newly elected members include Dr. Anne Stone as President‐elect, Dr. Ashley Hammond as Chair of History and Honors, and Dr. Kevin Hatala as Chair of Student Programs. See the Nominations Committee report for more information. These newly elected members of the Executive Committee were verified by the Executive Committee during their February 26, 2024, meeting.

### 1.12 Liaisons

Early Career Liaison, Dr. Justin Lund's term on the Executive Committee ends after a year dedicated to supporting Tribal Members. He introduced and pushed through the revision of conference registration fees and the establishment of a new subcommittee of the Committee on Diversity: Native American and Indigenous Biological Anthropologists (NIBA). Student Liaison, Elise Adams completes her term after efforts to support students. Like Dr. Lund, she has been a valuable member of our group. We thank our junior colleagues for their hard work and look forward to continued engagement with both individuals.

Our new Early Career Liaison, Dr. Nicole Torres‐Tamayo, brings interests in open data and data sharing to the Association. In addition, incoming Student Liaison, Lucas Fannin, will be leading efforts to support student professionalization and communication between students and other AABA members. We are delighted to be joined by these remarkable early career colleagues.

### 1.13 Ethics

The President, with the concurrence of the Executive Committee, appointed a new Ethics Committee Chair, Dr. Yohannes Haile‐Selassie, to lead the committee in the coming years. Dr. Haile‐Selassie has been joined by newly appointed committee members Drs. Carlina de la Cova and Cecil Lewis. Additional members may be added in the near future.

### 1.14 Science Policy (the Use of Legacy Data From Skeletal Collections)

The new, reformed Science Policy Committee is tasked with developing conversations around the use of historical datasets collected from legacy skeletal collections. The chair of this ad hoc committee is Dr. Lumilla Menendez. Nominations are under consideration for additional committee members.

### 1.15 Task Force on the Ethical Study of Human Materials

This Task Force was initiated by then‐President Steve Leigh in 2022. Co‐Chairs Fatimah Jackson and Ben Auerbach report that the Task Force is moving towards completing its charge this year. Recommendations will be presented to AABA leadership this spring and the Executive Committee will work on how to implement the recommendations.

### 1.16 Web Resources Upgrades

The AABA has made significant progress on revising web and conference resources. Our website, bioanth.org, was developed many years ago and is administered by Dr. Ed Hagen. The website includes conference resources to handle submission and review of abstracts, along with program and abstract issue production. These resources have served very well in the past. However, as we have moved towards a wider range of formatting for the annual meeting, we have begun to lean on commercially available platforms. These platforms offer new resources and provide flexibility beyond our traditional conference systems.

In 2022 and 2023, a committee chaired by Dr. Anne Grauer carefully evaluated our resources and recommended the revision of both bioanth.org and conference capabilities. Soon thereafter, President Leslea Hlusko charged a committee to continue the work of Dr. Grauer's committee, with the goal of implementing new web and conference resources by the end of April, 2024 so that our 2025 conference cycle can utilize new resources. The committee is chaired by Past and Acting President Steve Leigh, with members Anne Grauer, Ed Hagen, Catherine Taylor, Kristi Lewton, Amy Rector, along with Heide Rohland and Lori Strong (Burk and Associates).

This committee identified several priorities, including updating and improving our current web presence for users, taking advantage of commercial conference software, and developing resources for membership administration. Membership administration is currently managed by a third‐party vendor who will soon retire. The group met virtually through the year discussing and evaluating options, including retaining or substantially revising our current resources. We consulted with two comparable associations (e.g., the Society for Integrative and Comparative Biology and the Semiconductor, Environmental, Safety, and Health Association), both of which had recently undertaken similar initiatives.

After evaluating proposals from multiple vendors for our website, conference software, and membership administration, the committee moved forward with proposals from two companies to complete this work. Specifically, the committee accepted proposals from Knockmedia to develop a new version of bioanth.org. We chose X‐CD to handle conference tasks and membership administration. It is important to note that we worked with X‐CD for our 2023 conference in Reno to handle virtual components. X‐CD is also presenting our 2024 program, both for LA and virtual components.

Contract costs were $36,000 for Knockmedia and $10,500 for X‐CD. Costs for Knockmedia are one‐time only, with the option to pay for future consulting and services. The X‐CD contract includes per capita costs for membership administration, which we expect will total about $1250 per year. In addition, the X‐CD contract for 2025 will be $18,000, with AABA using the system beginning immediately after our 2024 conference. Our current costs for these services are approximately $6000 per year, so the change to a commercial conference software brings a cost increase. We are on track to complete the move to new web resources as our 2024 conference concludes.

A special thank‐you is extended to Acting President Steve Leigh for chairing this extraordinary committee, ushering in the new web‐based infrastructure for AABA's annual meeting and membership needs, and the massive redesign of the Association's website.

### 1.17 Contract Renegotiation With Burk

The Association will continue to work with our business partner, Burk and Associates (BAI), following contract negotiations in the late spring of 2023. The association has realized substantial benefits from our partnership with Burk and Associates over the last 7 years. The main benefit has been to relieve Local Arrangements Committees from risks, liabilities, and the large time commitments involved in negotiating contracts with venues and organizing the annual conference. In addition, we have benefitted by taking advantage of resources BAI offers to clients, such as webinar infrastructure, a more advantageous position from which to negotiate contracts, accounting and financial support, and deep knowledge of academic association best practices. The company also facilitates communication and interaction among clients, enabling us to take advantage of resources, ideas, and best practices from peer associations.

The cost of BAI's services will rise moderately from $126,000 to $150,000 per year. This will be the first price increase since the initial contract in 2017. The new contract will involve annual adjustments in line with the cost‐of‐living increase index published by the Federal Government for the preceding 12 months (CPI‐U), so the costs will likely increase each year. The contract also stipulates that changes in the size or complexity of the AABA (in either direction) can serve as a basis for altering fees. The contract is open‐ended, and can be terminated by either party at any time with a 1‐year notification, and will continue until terminated by either party, modified by an addendum as needed. The contract will be signed in person during the Los Angeles meeting and be effective as of January 1, 2023.

### 1.18 Statements

The Association either published or co‐signed a number of statements over the past year with the objectives of maintaining high scientific standards, supporting members and departments, and addressing social and political issues that directly relate to the research conducted within biological anthropology.

#### 1.18.1 National Anthropological Archives Support Letter (Co‐Signatory)

The AABA cosigned a letter in support of additional resources for the National Anthropological Archives (Smithsonian Institution) led by the Society for American Archaeology and joined by eight other professional anthropological associations and societies. The letter requests greater allocations of staff and resources to preserve the archives in accordance with professional standards. A copy is available upon request.

#### 1.18.2 Fossils in Space

The Executive Committee wrote a statement questioning the act of launching original fossil materials into space as an apparent publicity event (https://bioanth.org/about/position‐statements/aaba‐statement‐concerning‐the‐hominin‐fossils‐carried‐on‐virgin‐galactic‐spaceflight/).

#### 1.18.3 Trans Lives Support Statement

We joined AAAG, DAA, HBA, PPA, PAS, and the Biological Anthropology Section of the AAA in a statement in support of trans lives individuals. https://bioanth.org/about/position‐statements/aaba‐statement‐in‐support‐of‐trans‐lives/

#### 1.18.4 Position Statement to Marriott Regarding Labor Dispute

A copy is available upon request.

#### 1.18.5 President's Correspondence With University of North Carolina Greensboro

President Hlusko, in collaboration with AABA officers, submitted a support letter (dated January 22, 2024) to the Chancellor and Provost of the University of North Carolina Greensboro contesting the closure of the Department of Anthropology at the UNCG. Unfortunately, the department was discontinued as of February 1, 2024. A copy is available upon request.

### 1.19 Acknowledgments and Appreciation

This past year was an unusual one for the AABA presidency, with Leslea Hlusko's extended medical leave. The entire Executive Committee carried more work than would normally have been theirs in order to compensate for the absence. By far, Steve Leigh carried an especially heavy load. He dedicated an immense amount of time and energy to the Association, serving in both the roles of Past President and President simultaneously. On behalf of the entire AABA, Leslea Hlusko extends a huge thank‐you to Steve Leigh and to the Executive Committee for so graciously taking on so much more work than they signed up for.

Both Steve Leigh and Leslea Hlusko want to express immense gratitude to the entire Executive Committee for their dedicated service over the last year. It is an honor and a joy to work with such a talented group of colleagues, all of whom are dedicated to making AABA be its best.

We also extend a huge thank‐you to our colleagues at Burk Associates Inc. (BAI) who provide AABA with unfailing administrative support and guidance. We especially thank Lori Strong for her incredible dedication and hard work on all aspects of our complicated annual meeting planning. A special thank‐you also to Brett Burk, Jill Drupa, Lauri Mullins, and so many others at BAI who have proven to be highly valuable partners in carrying out the aims of AABA.

## 2 Report of the Vice President

Kristi Lewton, the Vice President and Program Chair, presented comments and submitted the following written report:

The Vice President chairs the Program Committee and coordinates AABA programming throughout the year. In 2023–2024, this centered on organizing the 93rd Annual Meeting in Los Angeles, California. This is the first time that AABA has held a conference in Los Angeles, and only the fifth time in California. We are very happy to be meeting in Los Angeles after our 2020 Los Angeles meeting was canceled due to the COVID shutdown in Spring 2020.

Tasks related to organizing the annual conference included program committee formation, symposium proposal review, abstract submission review and notification, workshop proposal review, webinar coordination, scheduling the conference events, publication of the annual abstract volume, and publication of the conference program (see Appendix 1 for the 2023–2024 timeline of these events).

The 2024 conference is in‐person with select virtual components. Online presenters have the opportunity to present in an asynchronous online format. This change in the online presentation options results from the feedback AABA obtained from Reno conference attendees via the “Feedback on AABA virtual conference events” survey in Spring 2023. The Invited Podium Symposia, Business Meeting, Presidential Panel, and select workshops will be livestreamed and recorded.

### 2.1 Program Committee

A call for program committee volunteers was distributed to AABA members in May 2023 with a deadline to apply by July 1, 2023. Eligible regular members applied by web form. The program committee includes 67 members, a mix of returning and new members. The committee includes members from a range of career stages and international institutions. Amber Jaeger served as the assistant to the Vice President. Program committee roster for 2023–2024 was: Donovan Adams, Francisca Alves‐Cardoso, Benjamin Auerbach, Shara Bailey, Miriam Belmaker, Michele Bleuze, Vanessa Campanacho, Stephanie Canington, Janine Chalk‐Wilayto, Carter Clinton, Siobhán Cooke, Maria Ana Correia, Miguel Delgado, Anthony Di Fiore, Nathaniel Dominy, Nicholas Ellwanger, Kori Filipek, Rebecca George, Halszka Glowacka, Mark Grabowski, Neysa Grider‐Potter, Elaine Guevara, Angela Harden, Amber Heard‐Booth, Megan Holmes, Genevieve Housman, Kent Johnson, Saige Kelmelis, Brittany Kenyon‐Flatt, Andrew Kim, Krystiana Krupa, Myra Laird, Ellis Locke, Christopher Lynn, Heli Maijanen, Hannah Marsh, Lumila Menéndez, Emily Middleton, Christina Nicholas, Heather Norton, Robert O'Malley, Marin Pilloud, Stephanie Poindexter, Luca Pozzi, Sean Prall, Kathryn Reusch, Michael Rivera, Gwen Robbins Schug, Joshua Robinson, Caroline Rowe, Sarah Schrader, Amy Schreier, Maja Šešelj, Michelle Singleton, Katie Starkweather, Sean Tallman, Christina Torres, Nicole Torres‐Tamayo, Catalina Villamil, Amelia Villaseñor, Cara Wall‐Scheffler, Kerryn Warren, Julie Wieczkowski, Amanda Williams, An‐Di Yim, and Chi Zhang.

### 2.2 Webinars

This year the Vice President recommended that the Executive Committee appoint an Associate Chair of the Program Committee to coordinate the AABA webinar series. Kevin Hatala was appointed to this position and solicited ideas for webinars.

The 2024 webinar series includes the following:

#### 2.2.1 Towards A Publicly Engaged Biological Anthropology (January 30, 2024)

The field of biological anthropology encompasses a range of fascinating but often socially contested concepts and issues such as human origins and evolution, genetics, race and racism, sex, gender and sexuality, and other dimensions of human difference. Some elements of the discipline, including common practices of fieldwork and local collaboration (or lack thereof), continue to reinforce harmful patterns of behavior grounded in colonialism, racism, and Eurocentrism. The United States and the world at large face a social climate of growing polarization and distrust of science and scientists, and (in some cases) active political censorship and sanction. Given these challenges, how can biological anthropologists engage with their students and diverse communities about the work of our discipline in ways that are inclusive, equitable, just, and impactful? This webinar will highlight examples of impactful education and public engagement activities by AABA members as well as voices from beyond our discipline. The session will include an open discussion as well as a breakout session for attendees to share and learn with other members. We will also invite attendees to share resources and reflections on a shared “Miro” board. This webinar is co‐organized with the AABA Education Committee.

Organizer: AABA Education Committee.

Panelists:

- Dr. Kathryn (Katie) Ranhorn (she/her), Assistant Professor, School of Human Evolution and Social Change & Research Scientist, Institute of Human Origins, Arizona State University
- Dr. Elaine Guevara (she/her), Lecturer, Department of Evolutionary Anthropology, Duke University
- Dr. Eshe Lewis (she/her), Project Director, Public Scholars Training Fellowship Program, SAPIENS Magazine
- Dr. Rob O'Malley (he/him) (moderator), Public Engagement Associate, Department of Genetics, Harvard Medical School & AABA Education Committee Co‐Chair

#### 2.2.2 Open Science in Biological Anthropology (April 10, 2024)

Our goal is to introduce attendees to the concept of Open Science, particularly Open Access, Open Methods, and the FAIR and CARE principles of data sharing. Participants will discuss the challenges and benefits they have experienced when employing Open Science practices in their research, and provide recommendations on how attendees can apply these practices in their own work, and what aspects should be considered. Further, with this webinar we hope to create more awareness of Open Science in biological anthropology and create a space where Open Science practitioners, both those who are experienced and those eager to learn more, can find each other and share experiences.

We also want to explore what more can be done by AABA and the biological anthropology community to encourage more open research in biological anthropology through a Q&A with attendees.

Organizers: Esther Plomp and Bjorn Bartholdy.

### 2.3 Invited Symposia

Proposals for invited symposia were due on August 15, 2024. The Program Committee received 15 invited symposium proposals, which were reviewed by both the Program Committee and the Executive Committee; 13 proposals were accepted. Accepted proposals include 6 podiums, 6 posters, and 1 online only (Table 1).

**TABLE 1:** Accepted invited symposia for the AABA 2024 meeting, their organizers, and format.

Invited symposium title	Organizers	Format
Beyond Bones, Bodies, and Behavior: Shifting the Disciplinary Landscape towards Biocultural Bodies	Andreana S. Cunningham, Isis Dwyer, Delande Justinvil	Podium
Central Asia: at the crossroads of Eurasia	Ainash Childebayeva, Guido Gnecchi‐Ruscone, Taylor Hermes	Podium
Terminology, Theory, and Method for the Study of Inequality of Health: Breakthroughs or Barriers?	Heather J.H. Edgar, Lexi O'Donnell	Podium
The growth and development of sub‐adult hominins: multidisciplinary approaches	Debra Bolter, Noël Cameron	Podium
Undermining the production of race science	Charles C. Roseman, James H. Jones	Podium
Women have always been here: Working towards a biological anthropology of women	Katharine C. Woollen, Kathleen D. Stansbury	Podium
60 years of studying human evolution: The ongoing contribution of Yoel Rak to Paleoanthropology	Alon Barash, Ella Been	Poster
Bridging Disciplines with Bone Biology	Victoria M Dominguez, Sophia Mavroudas	Poster
Endurance locomotion in humans: Diverse perspectives on long distance advantages	Cara M. Wall‐Scheffler, Martin Hora, Michal Struška	Poster
Kaye's Communities: Recognizing the Research, Teaching, and Mentorship of Kaye E. Reed	Amy L. Rector, Irene E. Smail	Poster
Rethinking primate and human origins: A symposium honoring Matt Cartmill	Natalie M. Laudicina, Aaron A Sandel, Erica Cartmill	Poster
William L. Jungers, a giant in the fields of paleoanthropology, size and scaling, and functional morphology: A commemorative tribute	Liza J. Shapiro, Laurie Godfrey, Christine Wall, Roshna Wunderlich	Poster
The Application of Artificial Intelligence (AI) in Biological Anthropology and Related Subfields	Mario Modesto‐Mata, Stephanie Canington	Virtual podium

### 2.4 Abstract Submission and Review

#### 2.4.1 Abstract Submission

The abstract submission portal was opened on September 15, 2023. Abstracts for the 2024 annual meeting were due October 15, 2023. A total of 782 abstracts were submitted, which is on par with the abstract submissions for the 2022 and 2023 annual meetings. However, these metrics still fall behind pre‐pandemic counts by ~30% (e.g., there were 1121 abstract submissions in 2019).

At the time of abstract submission, authors could choose to present in‐person or online only. Venue preferences for contributed abstracts at the time of submission were: in‐person 96% and online 4%. Compared to 2023, the interest in online only presentation has decreased from 11% of submissions to 4%.

#### 2.4.2 Abstract Review

Abstracts were assigned to two members of the Program Committee for review with a review deadline of October 31, 2023. Abstracts with one or more recommendations to reject received an additional round of review. After this second round of review a total of 8 abstracts had received at least two recommendations to reject, and these were rejected. All but one of these abstracts was rejected on the basis of a lack of evidence of data analysis or results (one was over the word limit). Authors of rejected abstracts were notified on November 8, 2023.

Authors of accepted abstracts were notified on November 11, 2023. Following the advance team visit to Los Angeles, California on December 8–10, 2023, authors of accepted abstracts were notified of the presentation schedule on January 4, 2024.

### 2.5 Workshop Proposals

We received 21 proposals for workshops by the November 15, 2023 deadline. This is twice as many as were received for the 2023 conference. Workshop proposals were evaluated by the chair of the Program Committee. All workshop proposals were accepted and organizers were notified on December 11, 2023. Two workshops were later withdrawn by the organizers. Two of these events are scheduled to be live streamed to the virtual platform (Table 2).

**TABLE 2:** Accepted workshops for the 2024 AABA meetings, the dates of their presentation, and their organizers.

Date	Workshop/panel	Organizers
20‐March	NSF Mock Review^a^	Rebecca Ferrell, Marta Alfonso‐Durruty, Lauren Schroeder
20‐March	CSViewer for Analysts – A big data approach to Cayo Santiago Rhesus macaque colony: A workshop on a software application to generate a user‐friendly interface and to appropriate data analytical tools	Qian Wang, Martin Zhao
20‐March	How to use B3GET: simulating your favorite primate in a virtual 2D environment	Kristy Crouse
20‐March	COD‐WIN: Negotiating Change in Professional Settings: A Workshop and Mentoring Opportunity for all Women AABA Attendees	Michelle Bezanson, Anne Stone
20‐March	Publishing in the American Journal of Biological Anthropology: Advice for Forensic Anthropologists and Bioarchaeologists	Tracy Prowse, Daniel Temple, Trudy Turner
20‐March	Trauma and thermal damage to skeletal elements: keys to identification and interpretation	Alison Galloway, Chelsey Juarez, Elayne Pope
21‐March	AABA Art, Culture and Science Engagement Exhibition	Michelle Bezanson, Robert O'Malley
21‐March	Anthropology Unleashed: Navigating Public Outreach in the World of Insta(ntaneous) Gratification	Sarah Reedy, Kelli Tamvada, Amanda Spriggs
21‐March	Departmental leadership and data: a discussion about trends in enrollment for current, past, and future departmental chairs	Amy Rector
21‐March	Improv for Anthropologists: Building Teaching and Outreach Skills with Applied Improv	Amanda L. Ellwanger, Marc Kissel, Natalia Reagan, Kimberly Congdon
21‐March	Microchimerism and human health: Bridging the gaps between anthropology and medicine	Amy Boddy, Kristine Chua
21‐March	Postdoc and Academic Job Hunting—Everything you need to know	Anna Ragni
21‐March	Up Goer Five: Communicating Biological Anthropology Using English's Ten Hundred Most Common Words	C. Kinley Russell, Briana Pobiner
22‐March	Evolutionary Mechanisms Beyond the Basics: Envisioning Complex Processes in Hominin Evolution^a^	Andrea Alveshere
22‐March	Backlash!?! Tools for Managing Trolls in Biological Anthropology	Jonathan Bethard, Michelle A. Rodrigues, Chris Stantis
22‐March	Considerations for Visualization and Documentation of Human Remains in Bioanthropology	Alexandria Orozco, Joe Kider, Lori Walters, Scott Branting
22‐March	Deconstructing the ‘Race as Biology’ Paradigm: The Role of Citations in Shaping Scientific Narratives and Practices	Ulises Espinoza, Agustín Fuentes, Rebecca Sear
22‐March	Queer & Trans Field Safety	Stephanie Meredith, Ellis Locke
22‐March	Sharing Your Science: Public Communication	Bridget Alex

^a^ Workshop is livestreamed.

### 2.6 Scientific Session Formation and Advance Team Visit

Scientific sessions were assembled by seven subcommittees of the Program Committee (Bioarcheology, Forensics & Dental, Functional Anatomy, Genetics & Education, Human Behavior & Biology, Primatology, and Paleoanthropology). Each subcommittee had 3–4 members (i.e., 25 members of the Program Committee were on subcommittees). Each subcommittee was tasked with grouping contributed abstracts into podium and poster sessions and assigning chairs to each session. The number of podium sessions per subject area was based on the proportion of abstracts in each subject area:

- Bioarcheology = 4
- Dental Anthropology = 1
- Education in Biological Anthropology = half session
- Forensic Anthropology = 1
- Functional Anatomy/Tissue Biology = 2
- Genetics and Genomics = 2
- Human Behavior = half session
- Human Biology = 2
- Paleoanthropology = 3
- Primatology = 3

Subcommittees were given a month to complete this task, and all sessions were formed by early December.

The AABA Advance Team visited the conference venue in Los Angeles from December 8–10, 2023. The team included Lori Strong (Burk & Associates), Eduardo Amorim, Steve Leigh, Kristi Lewton, Stephanie Meredith, David Raichlen, and Amy Rector. Because the sessions were formed prior to the advance visit, the team focused on scheduling sessions (assigning sessions to rooms, days, and times) and entering the sessions into the AABA meetings database.

### 2.7 Abstracts

**FIGURE 1:**
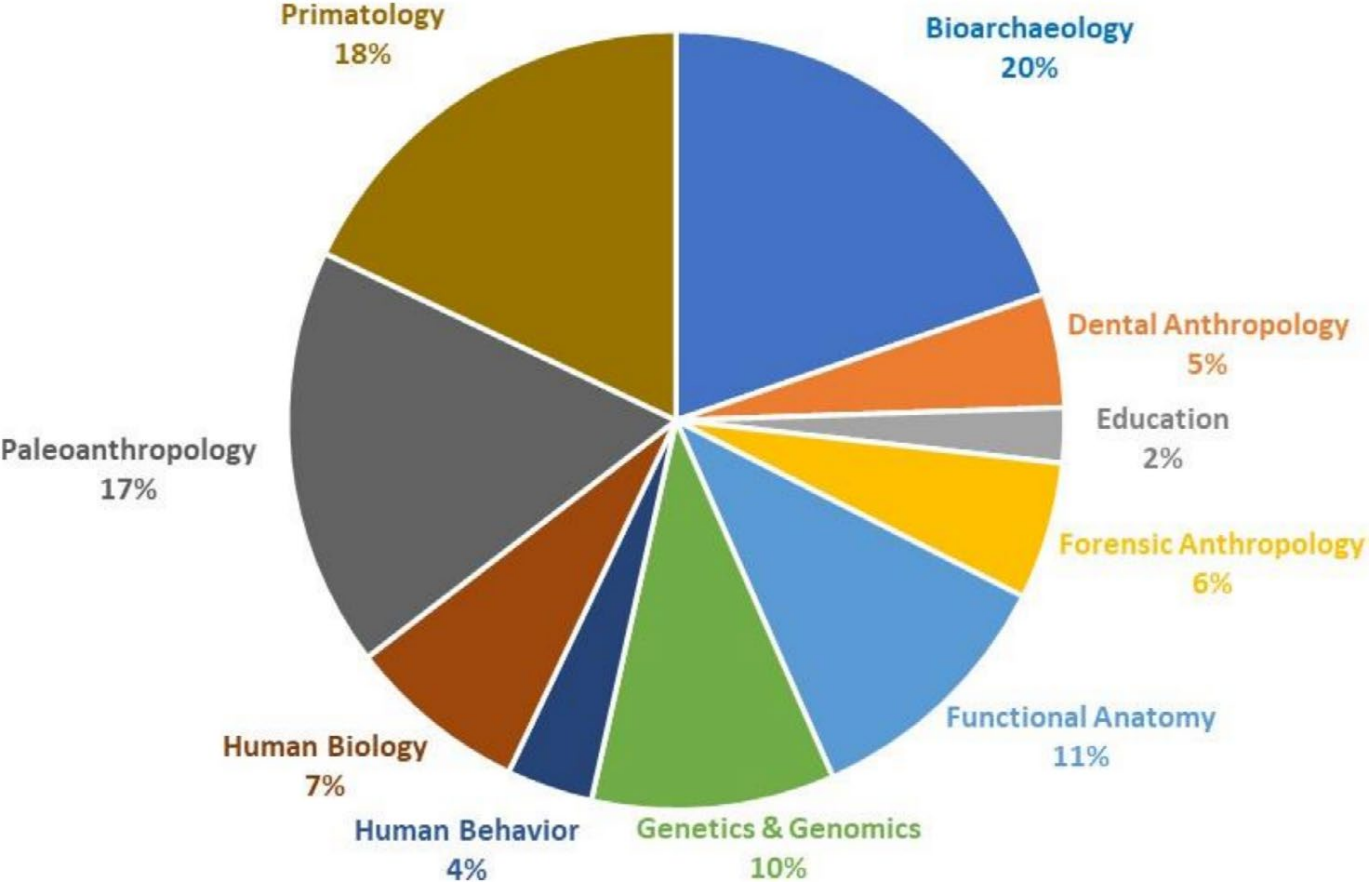
Proportions of contributed abstracts in each of the 10 major topic areas.

As of March 18, 2024, there are 612 contributed abstracts and 142 invited abstracts for a total of 754 abstracts. The proportion of contributed abstracts by subfield is in Table 3 and Figure 1. The abstract volume was submitted to Wiley on January 22, 2024. Proofs were approved on March 6, 2024.

**TABLE 3:** Number of contributed abstracts in each of the 10 major topic areas.

Topic area	Number of abstracts
Bioarchaeology	121
Dental Anthropology	29
Education	14
Forensic Anthropology	34
Functional Anatomy	67
Genetics & Genomics	62
Human Behavior	22
Human Biology	46
Paleoanthropology	107
Primatology	110

### 2.8 Meetings Program, Virtual Platform, and Accessibility

We are continuing to use the X‐CD platform for sharing virtual components of the conference, including as a repository for uploaded asynchronous online presentations and recordings of livestreamed sessions. These materials will be viewable through September for registered conference attendees.

The meetings program was finalized on February 21. The first draft of the full meetings program was published on the AABA website on February 21. Updates were published thereafter until the program was sent to the printer on March 6, 2024.

Following up on last year's efforts to increase accessibility of the annual AABA conference, we created a survey that accompanied abstract submission to query attendees on accommodation needs (e.g., oral presentation transcription, single level/no stairs). Of the surveys submitted by the abstract submission deadline, 0.5% requested an accommodation.

All podium presentations use voice‐to‐text transcription using PowerPoint subtitles and presenters have been reminded to use accessible fonts and color schemes. We have a quiet room with low lighting and reduced sensory stimulation, a Family and Lactation Respite Room, and access to a secure refrigerator for medications. The registration desk also has fidget spinners, eye masks, and ear plugs. All but one presentation room is setup as single‐level, without stairs.

### 2.9 Meeting Registration

As of March 18, there are a total of 1354 individuals registered for the 2024 annual meeting. International registrants from 37 countries make up 17% of meeting registrations, which is on par with meetings back to 2019 (Appendix 3).

### 2.10 Acknowledgments and Thanks

As my time as Vice President winds down, I want to thank all of the association members and leaders who help make this organization a welcoming, vibrant community. I am honored to have served the organization in this capacity and I am very fortunate to be both hosting (as a member of the Local Arrangements Committee) and organizing this year's conference in my home town. This year's program is outstanding and I am immensely grateful to the 67 dedicated members of our Program Committee who conducted and completed symposium and abstract reviews thoughtfully, thoroughly, and expeditiously. Additional thanks go to the 25 members of the Program Committee who took on the task of organizing the contributed sessions.

Many thanks to the Advance Team who met in Los Angeles to set the schedule for the scientific program. Thank you to Eduardo Amorim, Steve Leigh, Stephanie Meredith, David Raichlen, Amy Rector and to student volunteers Katie Beachem, Chloe Coulter, Cassie Croasmun, Alexandria Koedel, and Anamika Nanda. I also want to thank Michelle Bezanson for designing the beautiful AABA 2024 logo!

Of course, none of the program organization would be possible without the immense effort of Ed Hagen, our website and abstract database developer. The Program Assistant Amber Jaeger has been a tremendous help and I am very grateful for her outstanding organizational skills. I also want to thank the AABA Officers and members of the Executive Committee for their year‐round hard work that makes our annual meeting possible.

I thank our partners from Burk & Associates, including Annual Meeting Executive Director Lori Strong, Heide Rohland, Mary Lou Scarborough, Brett Burk, Cooky Bysura, Jill Drupa, Amy Sullivan, Sean Sullivan, Raelene Sok, Elizabeth Terry‐Humen, and Ruedi Birenheide. This team helped with registrations, organizing student volunteers, sending emails to the membership, producing our printed materials, interfacing with the exhibitors, and coordinating webinars.

Thank you to my fellow local arrangements committee members, Stephanie Meredith and David Raichlen, for helping to make the Los Angeles meeting unique and a success.

## 3 Report of the Secretary

The following report was submitted by Amy Rector.

### 3.1 Communications and Social Media

Communicating opportunities and events with AABA membership is a primary responsibility of the Secretary. For 2023–2024, our primary avenues of communication have included the website, email blasts through BAI, and AABA Twitter and Facebook. This year, we added a Bluesky account to the social media portfolio. We often receive feedback that our emails are spammed, and on all social media platforms engagement from membership has decreased from last year. We have 7900 Twitter followers, and we have seen a striking decrease in engagement (most Tweets are below 4% engagement for views), while the last 28 days leading up to meetings have averaged 1000 impressions per day. This means that only 1000 of our followers are seeing our Tweets per day. For the same time period on Facebook (with the same posts), posts reached a total of 5800 people and were engaged with 280 times. These metrics are reduced from last year. Adding images to Twitter and Facebook posts does not seem to improve their visibility or engagement.

### 3.2 AABA Webpage

An ad hoc committee dedicated to the development of a new web page was chaired by Steve Leigh and includes the Secretary and Webmaster Ed Hagen. New companies were chosen to host our membership and meetings management (XCD) and to develop the public‐facing website (KnockMedia). This integration of XCD and KnockMedia will be completed so that the new website can launch before the abstract submission portal opens for 2025.

### 3.3 AABA Executive Committee Communications and Digital Storage

With the assistance of Brett Burke of BAI, the Secretary initiated the adoption of a nonprofit status for our Google platform for streamlined storage of materials and centralization and continuity of email addresses as Executive Committee membership turns over. This shift from the current Dropbox storage system and unlinked emails is not yet complete.

## 4 Report of the Treasurer

The following report was submitted by Jonathan D. Bethard, for the fiscal year 2023:

This report from the Treasurer represents a preliminary account and assessment of the AABA finances between January 1st and December 31st, 2023. This report is preliminary because the accounting books for the 2023 fiscal year do not officially close until August 2024.^1^ By this point, however, most income and expenses for 2023 have been recorded.

At the close of 2023, the AABA showed a net operating cash loss of **$145,161.48** due to the normal expense of holding an in‐person annual meeting (Table 4). The AABA's total cash‐on‐hand for operational purposes was **$562,283.06** at the end of 2023 (Table 5).

**TABLE 4:** Operating gains/losses over 6 years.

Year	2018	2019	2020^a^	2021^b^	2022	2023
Income	656,313.02	704,526.51	461,730.55	338,720.64	465,264.17	461,381.43
Expenditures	899,209.55	850,920.38	409,715.61	300,165.50	551,503.85	606,542.91
Total	−242,896.53	−146,393.87	52,014.94	38,555.14	−86,239.68	−145,161.48

^a^ Annual Meetings canceled due to COVID‐19 pandemic.

^b^ Annual Meetings were held virtually due to the ongoing COVID‐19 pandemic.

**TABLE 5:** Cash‐on‐Hand over 3 years.

Date	12/31/2021^a^	12/31/2022	12/31/2023
Total	$415,813.27	$491,232.12	$ 562,283.06

^a^ Annual Meetings held virtually due to the ongoing COVID‐19 pandemic.

Fees for membership, meeting, and accounting services were provided by Burk & Associates and Gelman, Rosenberg, & Freeman. Accountancy services include our annual review and tax filing (available to any member by written request to the Treasurer), and bookkeeping. Other account expenses include credit card fees, Executive Committee expenses (including VP support, travel, and supplies), and legal fees to Allison, Slutsky & Kennedy. In calendar year 2023, the AABA also dispersed $62,221.05 in awards, including Cobb Professional Development Awards to support early career member research and Student Presentation awards to support student member research. Sources of income for 2023 are found in Table 6, and 2023 major expenses are in Table 7. A detailed Profit and Loss Statement for Fiscal Years 2020–2023 is included at the end of this report in Appendix 4.

**TABLE 6:** 2023 sources of income for AABA.

Source	Amount
Misc. Income (Paypal Deposit)	$343.00
Donations	$4568.00
Interest Income	$12,111.09
Wiley Contract Fee	$55,000.00
Membership Fees	$144,300.00
Annual Meeting Registration Fees	$245,059.34
Total	$461,381.43

**TABLE 7:** 2023 major expenditures for AABA.

Expense	Amount
Annual Meeting Costs	$304,460.04
Membership Servicing	$96,999.96
Awards	$62,221.05
Website Maintenance and Building	$40,750.00
Accounting	$37,152.25
Other Accounts	$65,959.61
Total	$606,542.91

### 4.1 Investments

The AABA's long‐term investments, managed by Merrill Lynch, gained over the previous year's losses. In 2023, our portfolio increased by ~15.7%: the net portfolio value at year‐end 2023 was $4,399,185.64, increasing from $3,803,745 on 12/31/2022. Investment funds are allocated between equities (in 2023 about 80% of our assets), fixed income (comprising 19% of our assets), and 1% cash. As always, the AABA works with our financial consultants to evaluate the allocation of funds to ensure that the proportion of funds in equity, fixed income accounts, and cash reflects the needs and goals of the AABA.

The AABA transfers 3.5% of its net investment portfolio (5‐year average) to our operating accounts on an annual basis. With this annual infusion into our operating budget, the AABA supports awards, grants, and programming during the annual meetings. In 2023, the amount transferred was **$137,590.00**.^2^

### 4.2 Summary

The AABA is a financially healthy organization, in part due to careful stewardship of our resources by previous Executive Committee leadership. The 2024 Annual Meeting in Los Angeles moves us a step closer to moving beyond contractual challenges related to the cancellation of the 2020 Annual Meeting due to COVID‐19. The 2026 Annual Meeting in Denver will be our last contractual link to pre‐COVID times.

Despite our current financial position, we will need to remain attentive to our annual finances given the consequences of the COVID‐19 pandemic, and currently, the US and global economies. At this point, however, the AABA remains a financially healthy association, and we aim to continue developing new initiatives and offering valuable programming for our members.

### 4.3 Acknowledgments

I owe a debt of gratitude and offer sincere thanks to the previous AABA Treasurer, Graciela Cabana, for helping me transition into the role of AABA Treasurer. There is a steep learning curve! Navigating QuickBooks is not an innate skill. I would also like to thank Anne Grauer, Trudy Turner, Steve Leigh, and Leslea Hlusko for their collegiality, especially related to conversations about maintaining the financial health of AABA. Thank you to Laurie Mullins, Lori Strong, and Tseme Turner (Burk & Associates) and Rob Clayton (Clayton & Associates) for their support and guidance during my time as AABA Treasurer.

## 5 Report of the Editor‐in‐Chief: *American Journal of Biological Anthropology*

The following report was submitted by Trudy Turner.

### 5.1 General Remarks

The *American Journal of Biological Anthropology (AJBA*) continues its 100+ year tradition of being a strong, vibrant and engaged voice for the biological anthropology community. Everyone associated with the journal, from editorial board members and associate editors to staff at Wiley, remain committed to providing the best possible science in a timely manner. This process is dependent in large part on our community—on all of you—who provide us with the reviews that allow the process to work. We all understand the task we are asking you to perform, the time it takes and the burden it is to perform this task. Everyone at the journal is incredibly grateful for your generosity and support. I continually marvel at reviews that exhibit both generosity and grace and I thank you for them. It ensures the strength of our discipline.

We were extremely pleased that we were able to publish two special issues of the journal this year. The first was “Evolutionary, ecological and biocultural perspectives on infectious disease and pandemics,” edited by Andrew Kim and Sabrina Agarwal. The second, recently released, was “A special issue in honor of the life and scientific contributions of Professor Mary Marzke,” a pioneer and innovator in the study of the evolution of the human hand, edited by Caley Orr, Tracy Kivell and Matthew Tocheri. We currently have four special issues that are in the process of reviewing articles and four additional special issues that will be accepting manuscripts in the very near future. We are very excited about all these topics and look forward to seeing them appear. If you have any suggestions for special issues, please get in touch with me.

**FIGURE 2:**
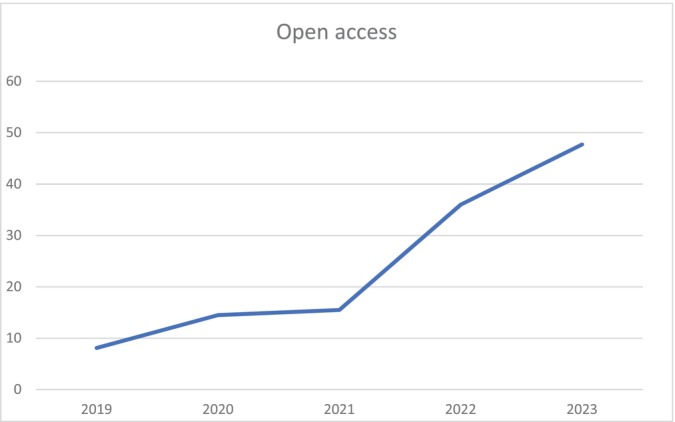
Rates of open access submissions to *AJBA* from 2019 to 2023.

One of the most exciting things for us has been the increase in Open Access manuscripts published (see Figure 2). Last year I reported that approximately one third of the manuscripts we published were open access and I set a goal of a 10% increase in OA articles a year for the rest of my tenure as Editor‐in‐Chief. I am very happy to report that we have far exceeded that goal for 2023. This year nearly half (48%) of the articles we published were OA. Almost all of these, 81 out of 84 articles, were able to take advantage of Wiley's transformational agreements. These agreements are contracts between institutions such as libraries, national, and regional consortia and publishers that transition the business model over time from one based on a paywall to one where publishers are paid for open access publishing services. Authors taking advantage of these agreements do not pay OA fees individually; they are paid for by the consortia at reduced rates. At present, Wiley has 79 transformational agreements with countries and institutions around the world. This number is increasing yearly. If you have a question as to whether your institution is covered, you can go to the Wiley website and submit a question. Please take advantage of this.

We would like to remind the community that an article must have a data availability statement in place to be published. This year the editorial board of the journal will discuss shifting from a model of “expects data sharing” to “mandates data sharing.” The difference between these models is that in mandates data sharing, the links to data in the data availability statement are checked to ensure that they work in the way the authors intend, and if data is shared in a repository, the data availability statement includes a permanent link to the data. As always, we welcome comments from our community. Please feel free to contact me if you have any comments on this policy.

This year we fully implemented the requirement for additional information on genetic studies using human subjects. The editorial board will review the results of the implementation of this requirement and its applicability to all studies using human subjects. At present, the implementation requires the actions of editorial board members and associate editors. We are looking into ways to make this more automatic for all submissions.

We try to offer a workshop at every annual meeting. This year, Tracy Prowse, Daniel Temple and I offered a workshop on Publishing in the *American Journal of Biological Anthropology*: Advice for Forensic Anthropologists and Bioarchaeologists. If you have any ideas for future workshops, please feel free to contact me.

### 5.2 Metrics

In 2023, the journal published three volumes (180–182), each of which had four issues. These do not include the two supplements: the annual meeting issue and the *Yearbook of Biological Anthropology*. Since there is effectively no longer a page limit to issues, Wiley no longer routinely reports the number of pages published. We received 352 submissions and published 176 articles. The acceptance rate is 52%. The number of submissions and the number of articles published both decreased by approximately 5%. This is consistent with all Wiley journals. All Wiley journals saw an increase in submissions in 2020 followed by a post‐Covid decline. Unlike many journals, the *AJBA* rebounded quickly and has remained relatively steady since the rebound. All of this points to the health of the journal.

The published content of the journal included: research articles (69.3%), brief communication (8.5%), media review (5.9%), technical note (4.8%), synthesis (3.6%), commentary (2.8%), letters to the editor (1.4%), reviews (1.1%) and other, including obituary, resources, and editorials (2.6%). The time from submission to first decision is 50 days. We are continually trying to find ways to reduce this number by setting short‐term reminders for editorial board members, providing multiple databases for accessing reviewers, and harmonizing decision terms, but we are limited by reviewer turnaround times.

**FIGURE 3:**
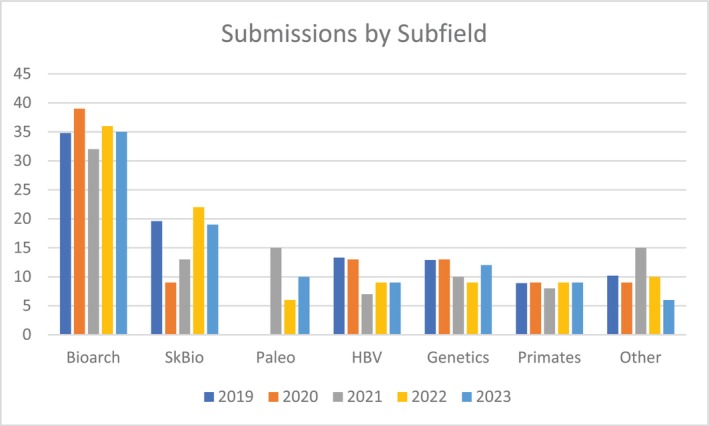
Submissions to *AJBA* by subfield from 2019 to 2023.

The number of articles we publish in the subfields of biological anthropology has remained relatively stable over the past 5 years (see Figure 3). For 2023, we published the following: Bioarcheology/Paleopathology/Forensics (36%), Skeletal Biology (22%), Paleoanthropology (6%), Human Biology (9%), Genetics (9%), Primatology (9%), and Other (10%).

The journal received submissions from 40 countries. Slightly over one‐third (35.3%) of submitted articles are from the United States. The countries with the greatest number of submissions are China, the United Kingdom, Spain, Brazil, Argentina, Italy, Australia, France, Germany, Canada, Japan, and Poland. Even though we had a significant number of submissions from China, we did not publish many of these articles. This seems to be primarily due to the articles being outside the scope of the *AJBA*. It is one of our goals to encourage international submissions that adhere to the aims and scope of the journal. We are hoping that our editorial board members can interact with colleagues to encourage appropriate submissions.

This year we transferred or referred 52 articles. If a manuscript is clearly out of scope for the journal, I can immediately transfer it to a Wiley service that will try to place the manuscript appropriately. If the manuscript is potentially of interest to the readers of the journal, I will confer with an Associate Editor and together we will decide whether to have the manuscript reviewed or referred to a journal with a cascading agreement. We can refer manuscripts to the *Yearbook of Biological Anthropology*, the *American Journal of Human Biology*, the *American Journal of Primatology*, *The International Journal of Osteoarchaeology*, the *Journal of Forensic Science* and the *Anatomical Record*. Half of the articles we refer or transfer are submitted elsewhere.

This year the *AJBA* has started reporting a number of additional metrics on journal performance. Wiley has endorsed the Declaration of Research Assessment or DORA agreement. The goal of DORA was to shift emphasis from a single journal‐based metric, such as the Impact Factor, to multiple other means of assessing research. To accomplish this, journals will greatly reduce emphasis on IF as a promotional tool, make available a range of article‐level metrics and encourage responsible authorship practices that highlight the specific contributions of each author. If you go to the *AJBA* homepage you will notice a series of additional metrics. We will still report IF. This year, there has been some confusion as to the Impact Factor of the *AJBA* that is tied to the change of the journal name. When the name of the journal changed from the *AJPA* to the *AJBA*, this change was filed and recorded with Clarivate, the organization that maintains the IF statistic. *AJBA*'s Impact Factor, under its previous title *American Journal of Physical Anthropology*, is 2.8 for 2022. As the journal went through a name change in 2022, the new title *American Journal of Biological Anthropology* has no 2022 Impact Factor, but the 2022 Impact Factor for *AJPA* is still valid, as it is the same journal. When the 2023 Impact Factors are released, there will be two values for the two titles as the citations and published articles are spread across both. All citations and published articles will be unified under the new title in the 2024 Impact Factor which will be released in 2025. The main take‐home message is that the *AJBA* maintains its IF rating and has never lost it.

One of the new metrics is Cite Score, which is administered by Scopus. The difference between the Impact Factor and Cite Score is that Cite Score is calculated on 4 years of citations instead of two. Almost all journals have a higher Cite Score than they do an Impact Factor. The *AJBA* Cite Score is 4.6. Last year there were 822,067 downloads of articles. This is approximately a 10% increase from 2022.

### 5.3 Acknowledgments

I am extremely grateful to the seven Associate Editors and 35 Editorial Board Members, eight Early Career Editorial Board Members, two Media Review Editors, and a digital fellow of the Journal. It is an honor to work with a wonderful group of dedicated, careful, insightful and caring individuals. This past year Claudia Valeggia, an Associate Editor, and Julia Fischer, an Editorial Board Member, both completed their terms. We welcomed Associate Editors Noreen von Cramon‐Taubadel, and Grazyna Jasienska; Editorial Board Members Susanne Cote, Hanya Goro, Jason Kamilar, Stanislaus Kivai, James Pampush, Dario Piombino‐Mascali, Zewdi Tsegai, Krishna Veeramah, Katherine Wander, Andrea Waters‐Rist, Qian Wang, and Molly Zuckerman; and Early Career Editorial Board Member Elizabeth Nelson to the board. I look forward to continuing to work with all of them. We will have some individuals completing their terms on the board in the next several months. We will be asking for applications for both editorial board members and early career editorial board members. We will publicize the call for applications and nominations on the AABA website and through social media. Please be on the lookout for the announcement and please apply.

I am also grateful to our publishers at Wiley. Gillian Greenough was recently replaced by Genevieve Richards. I very much look forward to continuing to work with Genevieve—she has been extremely helpful so far in navigating the many issues the journal faces. I am also grateful to Karthiga Pughalendhi, Xinrui Wang, Reeni Sunder and Tom Cannon for all their work for the office, production and special issues. I am mostly grateful for the biological anthropology community that graciously and generously gives of their time to ensure that the review process and the journal succeed. Thank you for the opportunity to edit the journal.

## 6 Report of the Editors‐in‐Chief: Yearbook of Biological Anthropology

The following report was submitted by Graciela Cabana and Sheela Athreya:

The 2023 issue of the *Yearbook* was Lyle Konigsberg's last issue as Editor‐in‐Chief, and we would like to thank him for his leadership. We also extend our gratitude to his editorial board for their service. Their tenure was particularly challenging as it coincided with the years of the COVID‐19 pandemic. Lyle managed to maintain the *Yearbook*'s publication on schedule and with the high‐quality articles for which it is known. We are looking forward to continuing that tradition as we work to put out our first issue as co‐Editors‐in‐Chief for 2024.

The 2023 issue featured seven articles:

- TL Kivell et al., *Form, function and evolution of the human hand*
- SA Williams et al., *African apes and the evolutionary history of orthography and bipedalism*
- T Tsutaya and N Mizushima, *Evolutionary biological perspectives on current social issues of breastfeeding and weaning*
- SS Urlacher, *The energetics of childhood*: *Current knowledge and insights into human variation, evolution, and health*
- DC Soto et al., *Genomic structural variation*: *A complex but important driver of human evolution*
- KS Paul et al., *Integrating genealogy and dental variation*: *Contributions to Biological Anthropology*
- BM Auerbach et al., *Morphology, evolution, and the whole organism imperative*: *Why evolutionary questions need multi‐trait evolutionary quantitative genetics*

The upcoming 2024 issue will be formally published in January 2025. We have confirmed contributions that will cover a broad range of sub‐areas within Biological Anthropology including Evolutionary Theory, Primatology, Paleogenomics, Bioarcheology, Forensic Anthropology and Human Biology. This issue is expected to have articles that address important theoretical orientations in our field including Black Thought in Biological Anthropology, and Power, Positionality and Lived Experiences within Communities. We have confirmed with invited authors that they will be submitting their manuscripts by May, and articles should be out in Early View by the Fall.

### 6.1 Editorial Board

We would like to thank our brilliant editorial board for their work since being invited to join 18 months ago. As we reported last year, we have changed the structure of the board from only advanced‐career scholars, to a mix of early‐ and advanced‐career scholars. We have paired early‐advanced scholars according to broad research specialties (human biology, paleoanthropology, primatology, bioarcheology, forensic anthropology, genomic anthropology, and ethics). We also invited scholars from institutions ranging from research‐intensive to community‐serving. Our editorial board (in alpha order) is Michelle Bezanson, Michelle Cameron, Habiba Chirchir, Zachary Dubois, Heather Edgar, Ann Kakaliouras, Ripan Malhi, Stephanie Meredith, Liv Nilsson Stutz, Alexandra Núñez de la Mora, Maanasa Ragavhan, Sean Tallman, Vivek Venkataraman, and Rachel Watkins.

At last year's AABA meetings in Reno, NV, the editorial board met for 3 h to introduce the new Associate Editors to each other, brainstorm ideas across sub‐areas, and identify contributors while at the conference. Gillian Greenough, our Wiley representative at the time, also attended the meeting to present a publisher's report. She has since been reassigned under Wiley's recent restructuring, but we want to publicly thank her for the support she gave us throughout our first year as Editors‐in‐Chief as we navigated the nuts and bolts of publishing. We are excited to be working with our new representative, Genevieve Richards, who is presenting the Publisher's Report at our board meeting this year.

The Editors‐in‐Chief began updating the Author Guidelines in 2023 because the current link on Wiley's page provides instructions for AABA articles only. We have clarified a number of issues related to length, content, formatting, etc., of the long‐form manuscript that is unique to *Yearbook* contributions. These updated Author Guidelines will be posted on the Wiley website in the late spring/early summer 2024.

*Yearbook* articles are typically by invitation, although we do consider volunteered submissions (auto‐submissions). Volunteered submissions will be evaluated according to their overlap and/or fit with topics that are being covered in the issue under planning, as well as how the article fits thematically with our vision outlined here: https://www.yearbookbioanth.com/ where we invite you to check for updates as well. If you have any questions about submitting to the *Yearbook* please contact us at yearbookbioanth@gmail.com.

## 7 Membership Chair Report

The Membership Committee report was presented to attendees in electronic form online, and orally at the Business Meeting by Stephanie Meredith. President Leslea Hlusko called for objections to new members. In the absence of objections by voting members in attendance, Membership Committee Chair Stephanie Meredith cast a unanimous ballot for the list submitted.

Stephanie Meredith submitted the following written report on March 10, 2024:

### 7.1 Membership Trends 2014–2024

Membership numbers and % yearly change presented in the table below reflect end‐of‐year totals, except for 2024. At the time of the 2023 AABA Member Report (3‐15‐2023), there were a total of 1375 members. This year's membership count (as of 3‐10‐2024) is up by 124 members, indicating an increase of 9% compared to a similar time last year. It is customary to report this metric. However, since 2017, the difference in total memberships from the time of the pre‐meeting data presentation to the end of the year has ranged from 80 (in 2020) to 574 (in 2018), and year‐to‐year differences in pre‐meeting membership numbers are more variable than year‐to‐year differences in end‐of‐year membership numbers (Table 8). Therefore, it is unclear whether a 9% increase in pre‐meeting membership numbers has any predictive value with respect to total membership trends.

**TABLE 8:** Membership trends: 2014–2024.

Year	2014	2015	2016	2017	2018	2019	2020	2021	2022	2023	2024
Count	1563	1950	2271	2263	2254	2074	1963	1474	1558	1572	1499
% change	+7.3	+24.8	+16.5	−0.4	−0.4	−8.0	−5.4	−24.9	+5.7	+0.9	n/a

### 7.2 Membership Outreach

In my 2023 report, I stated that low membership totals and indications that some regular members from 2022 had failed to renew for 2023 justified active outreach attempts aimed at rebuilding our membership. On January 31, 2024, I emailed all past members from any year since 2018 who had not renewed their membership for 2024 (1244 people) inviting them to renew their membership and to join us at the 2024 meetings. As of this writing, the number of regular members who have renewed for 2024 is nearly identical to the number of regular members who were paid through 2023 (+2).

### 7.3 Membership Demographics 2023

**FIGURE 4:**
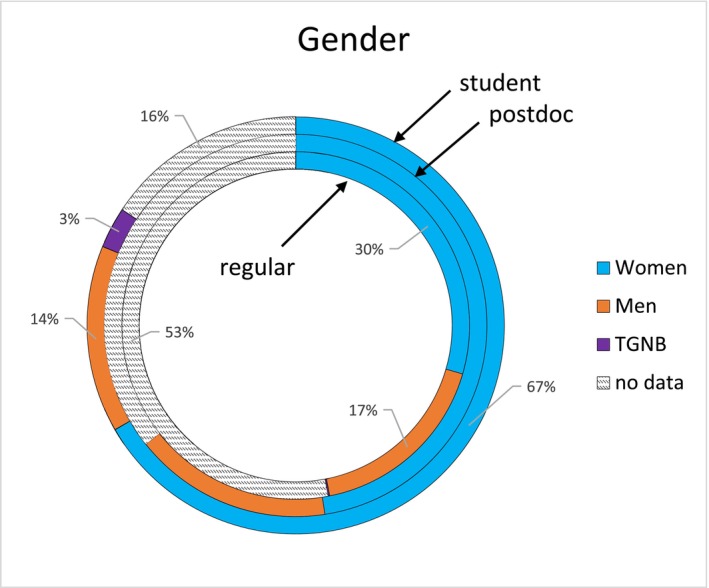
Available data on gender identity in the AABA membership. TGNB = transgender/nonbinary.

Changes were made to the 2024 membership renewal process to facilitate the acquisition of more complete demographic data on the membership. There was one programming error in that process (my apologies), which was corrected as soon as it was brought to my attention. I am hopeful that membership data collection and changes to membership data collection will be more streamlined in the new X‐CD system. This effort did not affect the 2023 member data, though, so the 2023 membership data still lacks gender data for 30% of members and ethnicity data for 51% of members (Figure 4). I am hopeful that 2024 members provided useful data during the renewal process and that next year's demographic reports will be data rich.

### 7.4 Membership Applications 2022–2023

Between March 15, 2023 and March 01, 2024, a total of 393 people newly applied for AABA Membership and were provisionally approved as new members (New membership applications prior to the 2023 report numbered 335). All new applications require approval by the membership (Table 9). During this time, 121 members were provisionally approved to transition to new membership categories. Some of these transitions can be approved by the Membership Chair and/or Executive Committee without a membership vote (Table 10).

**TABLE 9:** Applications for new membership.

Membership category	#	% of new memberships
Regular/Postdoc/Contingent (voting members)	85	21%
Special	19	5%
Student	289	74% (70% grad, 30% undergrad)
Total new members	393	

**TABLE 10:** Applications for changes in membership status.

Previous member category	New member category	Require vote	#
Postdoc/contingent	Regular	No	28
Regular	Retired	No	4
Regular (expired 5 years)	Regular	Yes	3
Special	Regular	Yes	2
Student	Postdoc/Contingent	Yes	34
Student	Regular	Yes	23
Student	Special	Yes	11
Special	Student	Yes	5
Uninteresting changes needing no vote		No	11
Total member changes			121

Most new applicants who applied for membership between March 15, 2023 and the start of the conference on April 19, 2023 were voted on by the membership at the 2023 business meeting even though their data were not included in the 2023 membership report; these names are not included in the list of names to be voted on in 2024 (Appendix 5), but their data are included in this report. Similarly, category change applications received between March 15, 2023 and the start of the conference on April 19, 2023 that required membership approval were voted on by the membership at the 2023 business meeting. The names of members whose membership changes do not require membership approval and/or who were voted on in 2023 are not included in the list of names to be voted on in 2024 (Appendix 5).

New and change applications continue to come in. Applicants who have been provisionally approved after data compilation for this report and who require membership approval have been added to the list of names to be voted on at this annual business meeting (Appendix 5) up until 3/17/2024, but their application data are not reflected in the summaries that have been presented above—their data will be included in the 2025 membership report. For these reasons, the numbers of names below will not match the numbers presented above.

## 8 Association Business

### 8.1 Constitution and By‐Laws Amendment Discussion and Vote

Procedure to revise Article III, Committees, Section 2b. Ad Hoc Committees: describes the responsibilities of the Local Arrangements Committee (LAC). Procedures required for the amendment of the by‐laws include an official written request by five AABA regular members for the amendment. A request to change the description of the Local Arrangement Committee was made in January 2023, the request was approved by the Executive Committee at the subsequent Executive Committee Meeting in February 2023, and the amendment was distributed in the 2023 annual business meeting. It was approved by a voice vote of the membership in 2023, distributed in the minutes of that annual business meeting, and the final voice vote was scheduled for 2024. After the proposed amendment was displayed at the 2024 annual business meeting there was no call for debate, and a voice vote was called. The motion was approved by a two‐thirds vote of the regular members present. The amended statement reads as follows:

Each annual meeting of the Association shall be supported by a Local Arrangements Committee appointed by the President, to serve until the conclusion of the annual meeting. The Local Arrangements Committee is responsible for supporting the Vice President and Program Chair with local matters related to the annual meetings. The President may, when necessary and with the consent of a majority of the members of the Executive Committee, appoint other ad hoc committees to deal with specific issues. Ad hoc committees exist for a period of up to three years. Additional years may be added by the President and Executive Committee.

## 9 Committee Reports

The AABA committee chairs provided written reports in electronic form in advance of the meeting (reports appear below), and the floor was opened for questions.

## 10 Nominations and Elections Committee

Report submitted by Chair of the Election Committee and Past‐President Steve Leigh.

The 2023–2024 Nominations Committee conducted the election of five Executive Committee positions. The committee consisted of Steven Leigh (Past and Acting President and Chair), and Drs. Anne Grauer, Dennis O'Rourke, and Karen Rosenberg. The AABA thanks these colleagues for their efforts.

Open positions included three officer roles (President‐Elect, Vice President and Program Chair, and Secretary) and two committee chairs (History and Honors and Student Programs Committees). Nominations were solicited through monthly AABA Newsletters and through contact with potential candidates, with self‐nominations encouraged. A web‐based form was opened in July to receive nominations. In addition, nominations could be made during the abstract submission process (as we have done for some time), with the deadline concurrent with the abstract submission deadline (October 15).

The nominations webform received a total of 10 nominations from July through October 15, with an additional 11 nominations submitted through the abstract submission system. Colleagues submitted several nominations directly to the committee. As of October 15, a total of 41 nominations had been received. The pool of nominations was diverse along numerous dimensions.

Several individuals were nominated for more than one position, and some individuals received multiple nominations for a single position. In all, 28 Regular Members were nominated for these positions (18 females, 10 males). Two candidates did not respond to correspondence regarding their nominations. Of the 26 remaining candidates, 10 females agreed to stand for election while 6 males pursued candidacies. Of candidates who chose to run, only two were available for president, three for vice president, two for secretary, with the remaining individuals running for either Student Programs Chair (4) or History and Honors Chair (5). Thus, the Nominations Committee was asked only to select three candidates for the committee chair positions, then forward these nominations to the Executive Committee to choose two finalists.

The Nominations Committee reviewed materials from each candidate, including a CV and statement. Three candidates for vice president and both chair positions were forwarded to the Executive Committee for final review. The Executive Committee chose the following candidates:

- **President‐Elect**: Lyle W. Konigsberg, Anne C. Stone.
- **Vice President**: Siobhán B. Cooke, Amy L. Rector.
- **Secretary**: Kristi L. Lewton, Julienne N. Rutherford.
- **History and Honors Committee Chair**: Nathaniel J. Dominy, Ashley S. Hammond.
- **Student Programs Committee Chair**: Kevin G. Hatala, Sean D. Tallman.

Voting opened on January 12, 2024 and closed on February 10, 2024. Turnout for the election was high, with over 200 votes for most positions. The results of the officer elections were President‐Elect, Anne C. Stone; Vice President and Program Chair, Amy L. Rector; Secretary, Kristi L. Lewton. For committee chairs, members elected Ashley S. Hammond (History and Honors Committee Chair) and Kevin G. Hatala (Student Programs Chair).

The AABA thanks all candidates. We look forward to working with our new Executive Committee members as their terms of office begin at the conclusion of our annual business meeting.

## 11 Professional Development Committee

Submitted by Lauren Schroeder

### 11.1 Early Career Liaison Program

We received three excellent applications for the Early Career Liaison program. The applications covered important topics including open science and data sharing, the ethical use of human skeletal data in teaching and beyond, and LGBTQIA+ community advocacy. Dr. Nicole Torres‐Tamayo (Postdoctoral Research Associate, University College London) was chosen as our new Early Career Liaison. In her application, Dr. Torres‐Tamayo details her advocacy work in open science, the importance of data sharing, but also the ethical concerns regarding the sharing of data from human individuals. During her tenure, Dr. Torres‐Tamayo will work closely with the Publications Committee, specifically Dr. Trudy Turner, to find mechanisms for retroactive data sharing in publications and to develop information‐ sharing initiatives on open science for early career scholars in Biological Anthropology. She will also be liaising with Dr. Yohannes Haile‐Selassie of the Ethics Committee on issues and concerns related to the sharing of data from human individuals. We wish her the best during her appointment as the AABA Early Career Liaison, and we thank Dr. Justin Lund for his service as outgoing Early Career Liaison.

### 11.2 Cobb Professional Development Grants (CPDG)

The AABA CPDG committee reviewed 25 applications for the 2023–2024 grant cycle. The applicants spanned multiple continents, with all subfields of biological anthropology represented. Overall, the pool of applications was very strong, reflecting the excellence of our early career investigators. Each application was reviewed by three or four committee members. In reviewing proposals, the committee considered the significance of the work, the quality of the proposal, the potential impact of the award on the applicant's career (including career stage and access to other funding sources), and the strength of the recommendation letter and CV. The committee recommended funding five proposals, up to $7500 each. Award and declination letters were emailed to applicants on March 6, 2024. The award recipients are as follows:

- **Victoria Dominguez**, Assistant Professor, Lehman College. *The Influence of Age on Porosity and Remodeling Across the Skeleton*.
- **Arwa Kharobi**, Assistant Professor, Masaryk University. *Experimental Study on the Osteocalcin Index to Detect Biochemical Markers of Stress in Human Bones (Hyksos, Egypt)*.
- **Kristen Savell**, Assistant Professor, Sacred Heart University. *Limb segment length, gait transition & cost of transport during slope‐walking*.
- **Melandri Vlok**, Lecturer, The University of Notre Dame Australia. *Investigating patterns of tropical disease with climate change in coastal and lowland populations in prehistoric Vietnam*.
- **Ashleigh L Wiseman**, Leverhulme Trust/Isaac Newton Trust Early Career Fellow, University of Cambridge. *Beyond the bones*: *Enhanced phylogenetic bracketing of hominin soft tissues*.

A committee of 22 AABA members, including the Chair, reviewed the pool of eligible proposals. I appreciate the time and effort that each reviewer provided in assessing these applications, and the helpful and constructive feedback given to each applicant.

In addition to the Chair, our committee this year was Benjamin Auerbach, Karen Baab, Andrew Barr, Elizabeth Berger, Michelle Bezanson, Michele Buzon, Sharon DeWitte, Celeste Gagnon, Anne Grauer, Lesley Gregoricka, Mark Hubbe, Jason Kamilar, Lyle Konigsberg, Elizabeth Miller, Cara Ocobock, Megan Perry, Marin Pilloud, Mary Silcox, Julie Teichroeb, Noreen von Cramon‐Taubadel, and Scott Williams.

### 11.3 Professional Development Panel/Workshop

The AABA Professional Development Committee and the Biological Anthropology directorate of the NSF ran a joint hybrid workshop on Wednesday, March 20, 8 a.m.–12p.m. at the 2024 AABA annual meeting in Los Angeles focused on a mock review exercise. In this workshop, early career scholars were introduced to the NSF merit review process by helping to review past senior NSF proposals provided by biological anthropology researchers using the NSF merit review guidelines. A panel of seasoned reviewers, Drs Christopher Schmidt, Andrea Taylor, Claudia Valeggia, Kieran McNulty, and Sharon DeWitte assisted workshop participants in the mock review of these proposals. The NSF program directors of the Biological Anthropology directorate, Drs Rebecca Ferrell and Marta Alfonso‐Durruty provided details of the merit review process. A portion of the workshop was also dedicated to discussing the development of a meaningful ethics statement.

## 12 Committee on Diversity of the AABA

Submitted by Susan Antón (Chair, AABA COD)

Equity, inclusion and community building are truly everyone's work. Although we report here on the formal work of the COD only, we do not mean to diminish the important work being done across the AABA by many constituencies.

The aim of the AABA Committee on Diversity (COD) is to increase diversity, equity, and inclusion within the field of biological anthropology. Established in 2006 and incorporated into the (then) AAPA bylaws as a standing committee in 2011, the COD is a consortium of member‐developed subgroups overseen by an appointed chair and a steering committee. There are now 10 (!) formalized interest groups.^3^ We encourage AABA members to join groups that interest them and/or develop missing groups. The overarching COD has a chair appointed by the AABA Executive Committee and a Steering Committee comprising at least one member from each of the interest groups. The subgroups are organized along standards that are developed independently by each subgroup.

The Committee on Diversity remains vibrant and generative. AABA COD is a “bottom‐up” organization and we are excited to recognize two new interest groups, COD‐SABA (South Asian Biological Anthropologists) and COD‐NIBA (Native and Indigenous Biological Anthropologists) who will hold their first events at the 2024 AABA meetings (more on this below).

### 12.1 COD Events at the 2024 AABA Conference

COD will host a number of activities at the 2024 meeting, both virtual and in‐person. Many are open to all attendees; a few are by invitation. Event details may be found in the subcommittee reports that follow.

- **Wed March 20th**—8:30–5 p.m. *COD IDEAS Student Scholars Workshop* (selection via application)
- **Wed March 20th**—2:00–5 p.m. *COD WIN Workshop*: *Negotiating Change in Professional Settings, a workshop and mentoring opportunity* (open to all)
- **Wed March 20th**—5–6 p.m. *COD WIN Happy Hour* (open to all; cash bar)
- **Wed March 20th**—6–8 p.m. *COD URS Undergraduate Research Symposium* (open to all)
- **Thurs March 21st**—8–10 a.m. *COD IDEAS Faculty Scholars Mentoring Meeting* (Faculty Scholars group members)
- **Thurs March 21st**—11 a.m.–12p.m. *COD Disability Organizational Meeting* (open to all)
- **Thurs March 21st**—5 p.m. *COD IDEAS & COD International Mixer* (open to all, cash bar)
- **Fri March 22nd**—10 a.m.–11 a.m. *COD NIBA Inaugural meeting of the nascent COD Native and Indigenous Biological Anthropologists subcommittee* (open to all)
- **Fri March 22nd**—12:15–2:15 p.m. *COD IDEAS Alumni Network Speed‐mentoring Event* (open to all program alums)
- **Fri March 22nd—**12:15–2:15 p.m. *COD AACT Subcommittee meeting and listening session* (open to all)
- **Fri March 22nd**—12:30–2 p.m. *COD LGBTQQIAA Business Meeting* (open to all)
- **Fri March 22nd**—2:15–4:15 p.m. *COD‐Disability*: *Nothing about us without us! A conversation on mentoring and being mentored for disabled scholars and allies* (open to all)
- **Fri March 22nd**—4–5 p.m. *COD‐SABA Chai Hour* (open to all)
- **Fri March 22nd**—4:15–6:15 p.m. *COD‐LGBTQQIAA Workshop*: *Queer and Trans Field Safety* (open to all)
- **Sat March 23rd**—8–9 a.m. *COD Steering Group—*(COD Subcommittee Liaisons)
- **Sat March 23rd**—9:15–10:15 a.m. *COD International Committee Meeting* (open to all)

### 12.2 COD Works with the AABA Executive Committee and the AABA Early Career Liaison

The COD regularly brings initiatives to the AABA executive committee and likewise serves in an advisory capacity when the President and board seek our input. To this end, the COD Chair attends executive committee board meetings as an ex officio liaison.

In 2023–2024, the AABA Early Career Liaison, Justin Lund, aimed to work with COD to address issues related to native and indigenous scholars in the discipline generally and AABA meetings in particular. Over the year, he worked with the executive committee on several topics. With VP Kristi Lewton, he worked to ask scholars to carefully consider each of the images they might use in presentations to contextualize potentially sensitive visual images. And Justin has envisioned the 9th COD interest group, COD‐NIBA, which will meet for the first time in 2024 to collectively consider future work and initiatives. Stephanie Meredith, AABA Executive Committee Member and COD‐LQBTQQIAA Liaison, along with COD‐LQBTQQIAA members took up important work in response to increasingly harsh legislation across the United States related to gender and sexuality. They worked with the executive committee to draft the AABA statement on Trans Lives and have work in progress on other issues related to gender and sexuality (see below).

COD Chair, Susan Antón, worked with the executive committee to update the AABA Officers Handbook, particularly related to COD process, protocol, and history.

### 12.3 Highlights of 2023–2024 Activities by COD Groups (Mostly in Order of Founding)

*NEW! COD‐NIBA (est. 2023; chair Justin Lund)*: The mission of COD‐NIBA (Native and Indigenous Biological Anthropologists) is to cultivate a vibrant and supportive community for the next generation of Indigenous biological anthropologists. Rooted in our shared commitment to preserving and advancing Indigenous knowledge, we strive to create a space that fosters collaboration, mentorship, and innovation. By nurturing a network of Indigenous researchers, we aim to amplify diverse voices, promote cultural sensitivity in anthropological research, and contribute to the empowerment of Indigenous sovereignties. Through collaboration, education, and advocacy, we seek to bridge the gap between traditional wisdom and scientific inquiry, paving the way for a future where Indigenous perspectives shape the forefront of biological anthropology.

*NEW! COD‐SABA (est. 2023; co‐chairs Sabrina Agarwal and Sheela Athreya)*: COD‐ South Asian Biological Anthropologists (SABA) aims to increase the participation and representation of students, scholars, and practitioners of biological anthropology who are of South Asian identity or descent, or who have South Asian community ties. We aim to offer professional support and camaraderie, and an organized voice on issues relevant to South Asian members of the AABA.

*COD‐IDEAS (est. 2006; co‐chairs Jada Benn Torres, Agustín Fuentes, Ripan Malhi, and Susan Antón)*: The new COD‐IDEAS NSF grant was funded (by PIs Graciela Cabana and Zaneta Thayer and Co‐Is Susan Antón and Ripan Malhi)!!! The programming extends and expands the original NSF‐funded COD‐IDEAS workshop (at the AABA meetings) for developing pathways into biological anthropology, adds regional in‐person COD‐IDEAS workshops in different parts of the country, and adds programming for faculty development through the career lifecycle. The AABA COD‐IDEAS Workshop will run all day Wednesday in advance of the 2024 meetings.

The first annual IDEAS Regional Meeting will take place in Nashville, TN on October 24–26th, 2024. Jada Benn Torres is the local host. Keep an eye out for the call for applications sometime after the AABA meetings!

COD‐IDEAS continues to staff an AABA booth at SACNAS, the STEM Diversity Conference (Sacnas.org). NYU supported faculty and student participation to attend and staff the booth in 2023. This has raised the discipline's legibility to STEM students.

At the 2024 AABA meeting in Los Angeles, IDEAS will reprise the events of AABA 2023, adding back a Wednesday workshop.

*COD‐URS (est. 2010; co‐chairs Cara Wall‐Scheffler and Marcie Myers)*: In 2023, we held our 12th Annual Committee on Diversity Undergraduate Research Symposium (COD URS). We accepted 49 posters representing 34 labs for the in‐person session. We had three presenters who only presented at the virtual symposium (noon PST on Wednesday, April 4), though a few of the in‐person presenters attended as well. Eleven of the 52 first authors were first‐generation college students, and at least 10 of the 34 labs were from programs that do not offer advanced degrees in anthropology. Across all the presentation options, we were able to support a total of 81 undergraduate students in their biological anthropology research. This symposium continues to offer a crucial opportunity for students to meet and talk with graduate students and potential graduate advisors. We had fantastic representation from across AABA elected officers (nearly all past presidents back to 2012!) and other senior members who came to support the students. In the virtual setting, we were also able to facilitate mentoring by graduate students who were unable to attend the in‐person meetings due to funding, including several international graduate students, making the COD URS an opportunity for lots of different kinds of students.

This year (2024) we will provide an in‐person symposium only, taking place on Wednesday, March 20 in Los Angeles. We currently have 66 posters, representing 50 labs from across the world. We further will have an international group of mentors, providing in‐person guidance and opportunities for each of the presenters. In total, we expect nearly 90 undergraduates to participate in this year's conference.

*COD‐WIN (est. 2013; co‐chairs Michelle Bezanson and Anne Stone)*: The subcommittee held virtual committee planning meetings and hosted an interactive panel discussion at the 2023 AABA meetings. The panel was titled: “Experiencing and negotiating the dynamics of power: Beginning the conversation.” This was a community conversation about experiences surrounding power dynamics, including a roundtable discussion about recognizing power imbalances and strategies for addressing them. Speakers included Sheela Athreya, Michelle Bezanson, Thierra Nalley and Anne Stone, and 22 AABA members at all career stages attended the workshop. It was a very successful event with most participants wanting more.

COD‐WIN includes committee members: Meghan Holms, Stephanie Poindexter, and Rebecca Rogers‐Ackermann as well as two new members, Thierra Nalley and Jessica Rothwell (graduate student member). Following the workshop, we have had several virtual meetings to discuss ideas for the 2024 COD‐WIN event.

*COD‐LGBTQQIAA (est. 2013; steering committee liaison Stephanie Meredith)*: COD‐LGBTQQIAA held their business meeting at the 2023 AABA meetings, during which time the group discussed the need for an Association Statement on Sex and Gender in response to increasingly harsh legislative efforts across the country to deny health care and personhood to transgender and gender non‐conforming people. Jonathan Bethard and Stephanie Meredith held a listening session on the idea of such a statement in September.

Samantha Archer, Zachary Dubois, Alexandra Kralick, and Rick Smith composed a Statement in Support of Trans Lives that was endorsed and published by the Association in November. Zachary Dubois and Stephanie Meredith are chairing the effort toward a Statement on Sex and Gender, which has been pared back to a “Primer on Sex (and sex‐based biologies),” and are currently soliciting interested authors to construct subdisciplinary writing teams.

Austin Lawrence and Chris Stantis continued to host “Bite‐size Bioanth” meetings on Zoom. Ellis Locke and Stephanie Meredith have organized a workshop for the 2024 AABA meetings on *Trans and Queer Field Safety*.

*COD‐AACT (est. 2013; chair Jessica Westin)*: COD‐AACT is regrouping. The subcommittee has an open discussion meeting at the AABA on Friday. If you identify with or are interested in any part of our acronym (non‐anthro department or teaching‐heavy positions, contingent or contract faculty assignments), please attend our meeting to discuss ideas for workshops, panels, sessions or anything else for future meetings and the AABA in general. Ideas may also be sent to current chair Jessica Westin.

*COD‐International (est. 2014; chair Rebecca Ackermann)*: COD‐I held a brainstorming meeting at the 2023 AABA meetings as well as a follow‐up virtual meeting later in the year. We will also be co‐hosting a mixer with COD‐IDEAS on Thursday evening, and holding a business meeting on Saturday morning.

*COD‐DISABILITY (est. 2022; chair Katie Kinkopf)*: COD‐Disability's mission is to foster belonging and provide a cultural space for disabled biological anthropologists (broadly conceived), to advocate for cultural change in the AABA with regard to disability as an intersectional and politicized identity, as well as devise long‐term, sustainable, accessibility recommendations to amend the existing AABA poster and presenter guidelines. At the 2024 meetings, COD‐Disability will hold an organizational meeting to recruit members, discuss future goals, and offer a supportive space for disabled and disability‐identified or allied members (Thursday 11 a.m.–12 p.m.). COD‐Disability will also sponsor a mentorship and networking event, *Nothing About Us Without Us! A conversation on mentoring and being mentored for disabled scholars and allies*. AABA members interested in joining COD‐Disability should reach out to Katie Kinkopf at kinkopf@berkeley.edu.

*Interested in starting another COD subgroup?* Please contact Susan Antón the COD Chair.

*Interested in joining a subgroup?* Please contact the subcommittee Chair or attend their events.

## 13 History and Honors Committee

The following report was submitted by Julienne Rutherford.

The primary business of the History & Honors Committee centered on three significant association awards: the Charles R. **Darwin** Lifetime Achievement Award, Gabriel W. Lasker Service Award, and the AABA and Leakey Foundation Communication & **Outreach** Award in Honor of Camilla Smith.

### 13.1 Awards

*Nominations and awardees*:

Total nominations = 14.

- Darwin, *n* = 8 (3 rollovers from previous year)
  ○ **Winner: Laurie R. Godfrey**
- Lasker, *n* = 2 (1 rollover from previous year)
  ○ **Winner: Steve R. Leigh**
- Outreach, *n* = 4 (3 rollovers)
  ○ **Winner: Tina Lasisi**

Approved rollovers for next year:

- Darwin, *n* = 3
- Lasker, *n* = 0
- Outreach, *n* = 1

Procedural issues:

- No new procedures were introduced this year. All procedures worked smoothly after the introduction of rank voting through google forms the previous years.
- For next year, I highly recommend the nominations be Google docs/forms: a one‐page nomination and disclosure forms for all signatories. No CVs or additional letters of support.
- The website states the nominations are a single page, and I emphasize this when I announce the opening of nominations via social media. Every year nominators reach out to ask if they can send more letters or other documents of support. I always say no.
- We had no disputed nominees this year.
  ○ As we've discussed in the past, disputes highlight the limitations of our disclosure forms and the need for Ex Comm members to carefully review the nominees and raise any concerns with the History and Honors Chair.
  ○ I suggest we also present the list of nominees to H‐CARE and Membership for review of past or current claims.
  ○ Awards are just that: awards. No individual is entitled to an award.
  ○ Does Ex Comm want to design and implement a formal process for removing a nominee from consideration? My view: What we did this year worked well, and with the additional layer of an external H‐CARE and Membership review I think is sufficient for the future.

### 13.2 AABA Oral History Project

No developments. Jon and Karen have not submitted any additional interviews.

### 13.3 Thank You

It has been an honor to serve AABA as Honors and History chair and to work with the members of the Executive Committee these last 3 years.

## 14 Student Programs Committee

The following report was submitted by Chelsey Juarez.

The Student Programs Committee primarily implements two student competitions. The first occurs at the annual meeting, when students who are presenting research in either a poster or podium format compete for seven named prizes. The second competition is traditionally for travel funds to attend the annual meeting, for which they write an essay on a topic chosen by the Executive Committee. In this report, I first summarize the 2023 student prizes awarded at the meetings in 2023, and finally, I present the 2024 travel awardees and preliminary information on the 2024 Student Presentation Prize Competition.

### 14.1 2023 Student Presentation Prizes

The deadline for entry to the 2023 AAPA Student Presentation was March 24, 2023, and will proceed following the protocol used for the last several years. We received an increase in student presentation prize applications this year compared to last year (*n* = 30) and 21 judges have been organized to evaluate these presentations (Table 11).

**TABLE 11:** History of the number of entries and judges for the Student Presentation Prizes.

Year	Number of entries	Number of judges
2014	39	—
2015	31	—
2016	45	21
2017	69	34
2018	63	39
2019	91	41
2020	53	—
2021	23	16
2022	21	11
2023	30	21
2024	15	27

AAA‐AABA Anatomy in Anthropology Prize

- **Telena Pounamu Hona**. *Understanding childhood growth*: *A pilot study of facial soft tissue thicknesses and body mass relationships in children aged 13–17 years*

AABA Prize Awards for Outstanding Student Presentations

- **Briana New**. *Considering the impact of somatic mutations on cranial structures*
- **Jordie Hoffman**. *The ecological and social context of women's hunting in small‐scale societies*
- **Audrey Arner**. *Sex differences in immune function and disease risk are not easily explained by an evolutionary mismatch*
- **Magdlena Palisson‐Kramer**. *Paleoenvironmental Reconstruction of the Okote, KBS and Upper Burgi Members of KoobiFora Formation* Via *Ecomorphology of Bovid Distal Metapodials*

Mildred Trotter Prize

- **Lily Hou**. *Reconstructing rodent locomotor behaviors using semicircular canal morphology*

Patricia Whitten Prize

- **Madelynne Dudas**. *A 3D geometric morphometric study of the central longitudinal axis of the radius across primates*

Journal of Human Evolution Prize

- **Alexandra Kralick**. *Queering Primate Osteology*: *Orangutan Skeletons Challenge Normative Assumptions of Binary Sex and Gendered Behavior*

American Association of Biological Anthropologists Honorable Mention for Student Presentations

- **Christoper Goden**. *Accuracy and Observer Agreement in the Determination of Trauma Timing in Human Ribs*
- **Megan Cole**. *Individual differences in baseline cortisol and cortisol reactivity among wild chimpanzees (Pan troglodytes) at Kanyawara*

Judges for this event were Chrisandra Kufeldt, Evan Garofalo, Mark Hubbe, Scott Maddux, Addison Kemp, Cris Hughes, Michael Rivera, Melanie Beasley, Jessie Goliath, Chelsey Juarez, Nicholas Passalacqua, Teresa V Wilson, Zaneta Marie Thayer, Jada Benn Torres, Eric Bartelink, Kelsey Ellis Melissa Clark, Jonathan Bethard, Katie Wolf, Elizabeth Cho, and Lori Trembly.

### 14.2 2024 Pollitzer Travel Awards

The Pollitzer Student Travel Awards are designed to help students defray the costs of attending the AAPA meetings. They are named in honor of William S. Pollitzer, a Human Biologist who taught at the University of North Carolina, Chapel Hill, a Darwin Lifetime Achievement Awardee, and past president of the AAPA. The number of awards is also tied to proceeds from the auction that is held at the annual meeting of the AAPA in the year prior. The award traditionally provides $500 to each recipient to defray travel costs to attend AAPA's annual meeting.

This award is open to all AABA student members (undergraduate and graduate) who are attending the annual meeting. Students do not need to be giving a presentation at the meeting to qualify but they do need to be a member of the AABA at the time of the meeting and should not have been granted their PhD prior to the submission deadline for 2024, the submission deadline was January 6, 2024.

The essay question changes each year. Awards are made on the basis of an essay of no more than 750 words (excluding references). The specific prompt for 2024 was:

The role of artificial intelligence (AI) in research and education has been a subject of discussion recently as the scientific community grapples with the widespread use of, for example, large language models such as ChatGPT in the classroom. Can AI be used ethically and in a way that is beneficial for multiple communities? Are there ways that it should not be used? How should we as a community move forward with artificial intelligence (should we restrict its use, provide guidelines etc.)? In your essay be sure to provide examples and address the ethical considerations of using AI in the biological anthropology spheres you inhabit (e.g., teaching/education, research/scholarship).

Evaluation was done on a 60‐point scale based on a five‐part scale (50 points total for originality and creativity, reasoning based on evolutionary biology and theory, and the current literature of our discipline, including references as needed; and 10 points for grammar and spelling). In an effort to standardize the scoring system, a rubric was utilized.

Each entry was given a number, and essays were identified only by number during the evaluation process. Each essay was scored by three judges. Each judge was asked to score ~8 essays and one back up judge was used for conflicts of interest. The final score for each essay was determined as the average of the three independent scores. Neither the named essays nor the number/name key was accessible at any time to the judges. No judge evaluated a proposal from a student at their same institution and the chair was not notified of any other conflicts of interest.

In order to implement the policy that priority would be given to novel entrants, students who had received a Pollitzer award previously had 3.5 points deducted from their final score. There were three entries from previous winners. Two of these ranked high enough to win a Pollitzer award again this year even with the penalty. There were 31 winners this year (Appendix 6), and submission numbers were lower than 2023 (Table 12).

**TABLE 12:** History of the number of entries and winners for the Pollitzer Travel Essay Competition.

Year	Number of entries	Number of winners
2012	—	43
2013	—	43
2014	—	50
2015	40	24
2016	68	42
2017	118	50
2018	75	57
2019	104	46
2020*	154	50
2021	31	28
2022	40	30
2023	87	30
2024	65	30

*Note:* *indicates year which moved completely online due to the pandemic.

## 15 Auction Report

Submitted by Shara Bailey and Madelynne Dudas

This year, we chose not to run a Live Auction due to decreasing engagement and conflicting scheduling. The Auction Committee reports the following for 2023: The Silent Auction brought in $8995. This is an increase compared to previous years.

## 16 Education Committee Report

Submitted by Drs. Kate McGrath and Rob O'Malley, co‐chairs (educommchairaaba@gmail.com)

*Local Engagement*. As part of the AABA 2023 Annual Meeting, six members of the Education committee staffed an engagement table at The Discovery (Reno's local science center) on the afternoon and evening of Wednesday April 19th. After the AABA meeting, the committee's cast collection was left with the AABA local arrangements committee for use by local educators who may wish to use it in classrooms or other science learning activities. We interacted with ~100 museum visitors (some of whom came specifically to meet us), and received very positive feedback from attendees and our hosts.

*AABA Meeting Programming and Activities*. In response to AABA leadership's call for funding requests at the 2022 AABA business meeting, we made a formal request for 3 k to support the committee's engagement activities. In 2023, we used this support to expand our traveling cast collection (including primate models and an adult male gorilla skull), and cover light catering for two workshops proposed or co‐proposed by committee members and aligned with the education committee's mandate: “Up Goer Five: Using Simple Language to Communicate Your Research to the Public” and “Improv for Anthropologists.” The committee also wishes to gratefully acknowledge the Leakey Foundation for providing honoraria for AABA members who are taking time out of the meeting to participate in outreach activities, and supporting printing and signage costs associated with our engagement. In addition to the Discovery outreach day, the committee piloted a small outreach effort at the AABA meeting venue itself (Peppermill Resort), exploring whether venue guests and staff might be an audience for public engagement at this and future AABA meetings. This was very well received, with many AABA attendees learning of the education committee's existence for the first time, and expressing interest in the website and associated resources. Finally, the Education Committee breakfast meeting held at AABA was used as an opportunity to share about the committee's work, connect with broader membership, and help to identify priorities for the committee in the months and years ahead.

*AABA Member survey*. In March of 2023 the Committee launched a survey for AABA membership to help identify needs, challenges, and opportunities for education and engagement in biological anthropology, to better guide the Committee's work. The survey has ~40 responses as of February 2024 and will remain open through the 2024 meeting in the hopes of garnering more responses (https://forms.gle/xsnuX3ughsV8Kbxu9).

*AABA Webinar*. Committee leadership organized a January 2024 webinar on “Towards and Engaged Biological Anthropology,” encompassing practices in fieldwork, education, and public writing. The webinar was recorded and will be posted on the AABA YouTube channel in the near future.

*Ongoing Activities*. The committee has hosted three virtual meetings since the 2023 AABA meeting. Meeting conversations have focused on ideas for workshops and other activities at the AABA meeting and website content. In particular we hope to see an array of new lesson plans for biological anthropology content added soon, and additional examples of great public science communication and engagement by AABA members. The committee wishes to thank Dr. Katrina Yezzi‐Woodley in particular for her work on the website. We have chosen to leave the survey instrument we launched in 2023 open, to capture additional responses at the 2024 AABA meeting about what members would like to see from the Education Committee.

### 16.1 2024 Meeting Activities

*Local Engagement*. The Education committee will host an exhibitor table as part of the Los Angeles County Natural History Museum's Homeschooler's Day (theme: Plants and People) on Wednesday March 20th. We will be part of an existing community program and anticipate engaging with 100s or 1000s of visitors.

*Exhibitor booth*. In light of the strong response to our pilot engagement at last year's AABA meeting, the Committee will staff an exhibitor booth this year to highlight the committee's work.

*Art, Culture, and Science Engagement Exhibition*. As a follow‐up to the extremely popular “un‐symposium” session at the 2023 AABA meeting, the Education Committee sponsored an exhibition at this year's AABA (in collaboration with Dr. Michelle Bezanson) to highlight science‐themed art, engagement and informal science learning activities by AABA members.

*Workshops*. Though not formally sponsored by the Education Committee, several members organized or co‐organized excellent workshops on engagement and education (including follow‐up sessions from last year on “Improv for Anthropologists” and the “Up‐Goer 5” challenge).

*Networking breakfast*. In lieu of hosting a formal Committee meeting at the AABA, we have chosen to host an education and engagement networking breakfast on Saturday morning to facilitate sharing and learning among AABA members (whether members of the committee or not). All AABA meeting attendees are welcome.

### 16.2 Future Plans

Our goals for the coming year include creating a more streamlined process for website content for the committee website, formalizing a process to develop Education committee‐sponsored workshops and activities at future AABA meetings, identifying ways for the Education Committee to support and promote AABA members in their independent teaching and engagement work, and exploring the possibility of virtual or in‐person programming year‐round. In particular, conversations at the 2023 AABA meeting highlighted a need for us to engage more intentionally with members and communities in the global South and marginalized and minoritized members of AABA. Anyone interested in the Education Committee should contact us at educcommchairaaba@gmail.com to be added to our email list.

Committee website: https://bioanth.org/about/committees/education‐committee‐page/

Active Education Committee members are defined as those who have attended at least one meeting or activity of the AABA Education Committee since the 2023 AABA meeting as of February 2024. Committee members include Kate McGrath, Rob O'Malley, John Mitani, Amanda Owings, Jacqueline Garnett, Amanda Ellwanger, Amanda Townley, Seth Chagi, Katrina Yezzi‐Woodley, Caitlin Schrein, Arielle Johnson, Kimberly Congdon, Kinley Russell, Briana Pobiner, Laura Newman, Natalia Reagan, Karol Chandler‐Ezell, Kristin Crouse, Christopher Schmitt, Malorie Albee, Natalia Reagan, Jessica Brinkworth, Kim Foecke, and Ryan McRae.

## 17 Harassment Committee for Awareness, Response and Equity (HCARE) of the AABA Report

Submitted by Andrea B. Taylor (Chair, AABA HCARE)

The HCARE was established by AABA Executive Committee leadership in February of 2020 as an ad hoc committee. The committee members serve by appointment of the AABA President. The committee is overseen by a chair, who is appointed for a four‐year term by the AABA President.

*Committee members*: Jessica Brinkworth, Agustín Fuentes, Stephanie Meredith, and Robin Nelson.

The HCARE was charged with the following responsibilities:

1. Assisting the Executive Committee officers in creating, implementing, and revising harassment, bullying, intimidation, retaliation, and/or assault reporting policies and procedures.
2. Receiving and responding to incoming reports of harassment, bullying, intimidation, retaliation, and/or assault.
3. Deliberating and determining appropriate responses.
4. Making recommendations to the AABA President.

The HCARE met six times between April 20, 2023 and March 20, 2024.

### 17.1 Complaints

The HCARE received and assessed nine complaints regarding concerning behaviors. Eight of the complaints were communicated to HCARE via NAVEX; one of the complaints was communicated via email. Eight of the nine complaints involved concerning behaviors that occurred at the 2023 annual meeting of the AABA. All nine cases have been resolved and are officially closed.

### 17.2 Activities

The Committee has been exploring ways of increasing awareness of the HCARE. Beginning at the 2024 annual meeting, HCARE “business” cards will be available that provide QR codes, the HCARE Gmail address, and a phone number, to both improve awareness of HCARE and to facilitate reporting of harassment and other concerning behaviors

### 17.3 New Committee Chair

At the close of the 2024 AABA business meeting, Andrea Taylor will rotate off as committee chair and Agustín Fuentes will assume the role of chair

## 18 Early Career Liaison Report

Submitted by Justin Lund

The early career liaison proposed several initiatives to make the AABA meetings more welcoming to Indigenous scholars, community members, and Tribal leaders; the proposal aligns with other anthropology associations. The proposed suggestions were (1) to create a special interest group specifically for Native American and Indigenous biological anthropologists, (2) provide a free or reduced‐rate fee for membership and conference registration for Tribal members, and (3) create communication to alert conference participants of human remains images.

1. The inaugural meeting of the Native American and Indigenous Biological Anthropologists (NIBA) has been scheduled for Friday, March 22, 2024 from 10 to 11:00 a.m. To facilitate a comfortable environment for discussion the meeting will be held offsite at Bluestone Lane Coffee
**Current NIBA agenda items**
  - What should we call ourselves?
  - Mission and vision
  - Organizing a special poster session or panel for AABA 2025
  - Reduced rate for Tribal members logistics
    - Who is a tribal member?
    - Committee to review Indigenous status
    - Storage/Confidentiality of information
  - NIBA meeting 2025
  - Human remains sensitives and possible solutions
2. Providing free or reduced rate fees for Tribal/Indigenous members was discussed with the Executive Committee. The ExComm suggested reduced rate fee similar to that of Special Member—qualifying country rate. For many, enrollment documentation could likely be provided as proof of Indigenous status. However, documentation may not be available for some who identify as Indigenous. Other organizations have accepted narrative submissions describing this lack of documentation. In consultation with one organization (American Association of Anthropologists), it was suggested that a committee of 3 or more people evaluate documents and narratives. It was also strongly encouraged that a confidentiality policy be put into place for tribal affiliation documentation and narratives. NIBA will discuss our thoughts and provide recommendations to the Executive Committee.
3. To address human remains sensitivities language was added to the abstract submission portal to encourage presenters to be thoughtful of their language and imagery. “We ask scholars in all subdisciplines of biological anthropology to please carefully consider the language used and images chosen to describe your research. Offensive language and images should not be used. In addition, please be mindful of whether the language in your presentation may inadvertently contribute to marginalization of underrepresented groups in biological anthropology. Please consider each image carefully to avoid unintended harm and if an image is essential but may cause difficulty for some viewers, please consider contextualizing or alerting audience members in advance.” NIBA will discuss human remains sensitivities and provide feedback and recommendations to the Executive Committee.

## 19 Student Liaison Report

Submitted by Elise Adams

*Committee Structure*. As per the structure created by the Executive Committee of the American Association of Biological Anthropologists (AABA), the *ad hoc* Student Committee co‐chairs are the current and incoming Student Liaisons for the AABA Executive Committee. The current (and outgoing) liaison is Elise Adams. The incoming liaison is Luke Fannin.

### 19.1 2023–2024 Student Liaison Activities

- The ninth annual AABA Student Meet and Greet is scheduled for Wednesday, March 20, 2024. It will be held in Georgia I of the JW Marriott LA Live. In consideration of the limited virtual setting of the 2024 conference, there is not an additional virtual Student Meet and Greet currently scheduled.
  ○ The eighth annual AABA Student Mixer was held in‐person during the 2023 AABA annual meeting in Reno, NV, and organized by the 2022–2023 student liaison, Dori Kenessey. The mixer included a networking activity of “find someone who”, with students who completed the activity receiving a small award in the form of a sticker with the 2023 AABA conference logo.
- The Student Liaison worked with the 2022–2023 Student Liaison (Dori Kenessey), and Executive Committee's Vice President (Kristi Lewton) and Secretary (Amy Rector), to update and improve the Student Resources page on the AABA website. The existing information was updated with working and additional links. The Student Resources page now contains a comprehensive list of links, organized into categories including Academic Writing and Publishing Resources, Professional Development Resources (how to write a CV or cover letter, etc.), Internship Opportunities, Teaching Resources (how to write a syllabus, tips for teaching assistants, etc.), Graduate School Resources (how to choose a school/advisor, balancing mental health and work, etc.), Fieldwork Resources, Funding Resources (internal and external), and Communities of Interest (i.e., other organizations that AABA Student Members might be interested in—Paleopathology Association, American Society of Primatologists, the American Academy of Forensic Sciences, etc.).
- Following the suspension of the AABA Student Members Facebook group, and other student programs and efforts established pre‐COVID which have not been restarted, there have been discussions of the best methods/forums to reestablish communication between the student liaison and student members, as well as facilitate discussion among student members. Social media sites (including Instagram, Threads, etc.), as well as former initiatives (a Room Sharing group, the Buddy Program, the Student Newsletters, and the ad hoc Student Committee meeting) should be evaluated for creation or reinstatement in future years.
  ○ A public Google form for finding a roommate for the 2024 AABA conference was made available on the AABA website (exact numbers and success rate not reported here).

### 19.2 Planned 2024 AABA Meeting (Los Angeles) Student Committee Events

- AABA Student Meet and Greet: Wednesday 5–6 p.m., March 20

## 20 Old Business

Renewal of contract with Burk & Associates Inc.

## 21 Reports by Affiliated Organizations

### 21.1 National Science Foundation (NSF) Program in Biological Anthropology, Division of Behavioral & Cognitive Sciences, Directorate of Social, Behavioral, & Economic Sciences (SBE)

Presented by Dr. Rebecca Ferrell, Program Director, Biological Anthropology.

- Incoming rotator: Marta Alfonso‐Durruty, KSU
- Recent solicitation requirements
  ○ ethics statement
  ○ data sharing timeline
  ○ additional vertebrate animals guidelines
- Recent solicitation opportunities
  ○ scholars proposals
  ○ community‐engaged research
  ○ research experiences for post‐baccalaureates

### 21.2 The Leakey Foundation

Presented by H. Gregory,

- Congrats to our executive director for 20 years, Sharal Camisa
- Joan Cogswell Donner Field School Scholarship—up to $2000 towards field school tuition
- Carbon Offset, are certified carbon neutral, including research in their calculation
- Evolution Exchange: young professionals group. Contact evolutionexchange@leakeyfoundation.org

### 21.3 Biological Anthropology Section (BAS‐AAA)

Presented by Robin Nelson

- 2024 meeting in Tampa
- Jada BT and Adam Van Arsdale are the program chairs—contact them. Submission window open and will close on April 26.
- Theme
- is praxis

### 21.4 American Association of Anthropological Genetics

Presented by Christopher Schmitt

- 85 members
- Education activities: App of Genomics to Anthropological Research (AGAR) workshop for 2024: Microbiome Workshop
- Networking event at AABA 2024
- New Editor for *Human Biology*: Connie Mulligan. IF 1.371
- New website
- AnthGen.org/Education. AAAG Anthropology and Society Resource List
- Student Awards

### 21.5 Wenner‐Gren Foundation

Presented by Danilyn Rutheford.

Overview of history and grants

- Postdoc fellowships
- Wadsworth fellowships
- Symposia that are interdisciplinary
- Global Initiative Grants—current theme is capacity building for paleoanthropological work in eastern Africa by eastern Africans

### 21.6 American Association for the Advancement of Science (AAAS), Section H, Anthropology

Submitted by George R. Milner, Section H Secretary

I should start by thanking Amy Rector for inviting me to report on Section H (Anthropology) of the American Association for the Advancement of Science (AAAS).

This past year has seen many changes in the AAAS as it transitions to a new governance structure. In 2023 and 2024, we are continuing to see evidence of growing pains, but the result will be well worth the considerable effort that has been involved in the governance changes, from the highest levels of the organization down to individual sections. The intent of new governance structure—and I understand it is the first such thorough change in over 70 years—will be to ensure more transparency in the organization's functioning, to foster a more inclusive environment, and to better represent today's diverse scientific workforce. Biological anthropology has been well represented in this process, notably by Karen Strier who for several years was heavily involved in the planning that led up to what is happening today.

One of the most notable changes for section members is the expanded group of section officers. Appointed positions during the first year of the transition have been replaced, in part, by elected officers. This process will continue until officer terms are staggered so there will be continuity over time in personnel on each section's Steering Group. The AABA is well represented in the Section H Steering Group for 2024. Anyone interested in running for office should contact Denise Su (Chair) or me as soon as is convenient.

I am pleased to say that 10 new Fellows from Section H Fellows were elected this past year, with AABA members being among them. Our 2024 Robert W. Sussman Award—presented at the AAAS annual meeting last month—went to Robin G. Nelson of Arizona State University. To quote from the announcement, she “exemplifies the spirit of the Sussman Award for her outstanding contributions to understanding human relationships and their evolutionary impact. She is a leader in efforts to advance equity and redress historical harms in the field of anthropology.” The section is pleased that the outstanding achievements of so many of our AABA colleagues have been recognized over the past year.

Perhaps it is best to close by saying that next year's AAAS meeting will be in Boston in mid‐February. I'm sure we can all agree with the sentiment expressed in the aspirational theme of the Boston meeting: Science Shaping Tomorrow. While it might not be the best time of year for air travel, I look forward to seeing many of you there.

### 21.7 New Business

- Discussion of AABA support for CONICET, Argentina's National Research Council.
- The Local Arrangements Committee for Baltimore, Maryland, 2025 shared information on the venue and site (Siobhan Cooke, Matthew Ravosa, and Adam Sylvester).

## 22 Remembering Members and Friends of the AABA

The *in memoriam* report was prepared by Past‐President Steve Leigh. Colleagues recognized included Christophe Boesch, Kimberlin C.K. “Bob” Brain, Frans de Waal, Eugene “Gene” Giles, Brian E. Hemphill, Ann L. Magennis, Sally McBrearty, Masatoshi Nei, Roy J. Shephard, Karen L. Steudel‐Numbers, John A. Van Couvering.

## 23 Resolutions

- We are thankful that our president is fully back in her role.
- We resolve to conduct our research, teaching, and service at highest professional and ethical levels.
- Resolve to support our members, especially the most vulnerable among us.
- We thank our LAC and look forward to working with our colleagues in Baltimore.
- Thanks to our outgoing Executive Committee members for their remarkable service through their terms and welcome our new members.
- We thank our business partners at Burk and Associates, especially Lori Strong and Mary Lou Robinson for helping us conduct a remarkable conference.

The meeting was adjourned at 9:07 PM Pacific Daylight Time by Leslea Hlusko.

## Author Contributions

**Amy L. Rector:** writing – original draft (equal).

## Data Availability

Data are available on bioanth.org.

## Endnotes

^1^
Since 2017, AABA finances have been tracked using an accrual accounting system. Unlike a cash‐based system, income and expenditures are recorded when incurred, meaning that transactions are recorded when they are made, regardless of when they are paid. For example, income generated in the Fall of 2020 for 2021 membership would not be considered 2020 income; the recording of this income is deferred until 2021.

^2^
Starting in 2020, the AABA began transferring 3.5% of its investment income into its operating account on an annual basis.

^3^
Committee on Diversity Increasing Diversity in Evolutionary Anthropological Sciences (COD‐IDEAS); COD Women's Initiative (COD‐WIN); COD Undergraduate Research Symposium (COD‐UGRS); COD LGBTQQIAA interest group; COD Anthropologists Outside of Anthropology Departments, Contingent, and Teaching Focused Faculty (COD‐AACT) Task Force; COD International (COD‐I); COD Transforming Anthropological and Scientific Knowledge (COD‐TASK); COD Disability; COD Native and Indigenous Biological Anthropologists (COD‐NIBA); COD South Asian Biological Anthropologists (COD‐SABA).

## Appendix 1 2023–2024 Timelines

**Table:**

Event/process	Open date	Close date	Reviews complete	Notification date
Program Committee Formation	5/25/2023	7/1/2023	NA	7/3/2023
Symposium proposals	7/15/2023	8/15/2023	8/31/2023	9/2/2023
Abstract submission	9/15/2023	10/15/2023 deadline, 10/18/2023 assigned to reviewers	10/31/2023	11/8/2023 (reject), 11/11/2023 (accept)
Workshop proposals	10/15/2023	11/15/2023	12/1/2023	12/11/2023
Presentation schedule				1/4/2024

## Appendix 2 2023–2024 and Historical Meetings Metrics

**Table:**

	2018	2019	2020	2021	2022	2023	2024
	Austin	Cleveland	Los Angeles (canceled)	Baltimore	Denver	Reno	Los Angeles
Registrations	1901	1802	NA	1079	1281	1363	1354
Abstracts submitted	1229	1121	1247	458	785	775	779
Abstracts accepted	1214	1090	1226	457	776	772	771
Symposia (submitted/accepted/presented)	26/26/24	23/22/21	26/25/24	8/8/8?	13/13/13	13/13/13	15/13/13
Podium symposium sessions	8	7	6	NA	6	5	6
Poster symposium sessions	16	14	18	NA	5	6	6
Online symposium sessions	NA	NA	NA	8	2	2	1
Contributed sessions	51	41	46		85	58	46
Podium sessions	22	18	23	NA	18	21	24
Poster sessions	29	23	23	NA	18	20	22
Evening poster sessions	2	1	0	NA	0	0	0
Online sessions	NA	NA	NA		49	16	1
Withdrawn submissions (as of 3/18/2024)	30	16	11	5	17	24	17

## Appendix 3 International AABA Registrants by Country, 2019–2024

**Table:**

Country	2019	2021	2022	2023	2024
Albania	1			1	1
Argentina	1	5	4	3	
Australia	6	7	7	8	6
Austria	2	4	5	4	8
Belgium	4	2		3	
Brazil	16	6	4	3	1
Canada	74	51	34	53	41
Chile	1	1		2	1
China	23	2	1	3	10
Colombia	2	1		4	
Croatia	3	1		4	
Cyprus	1				
Czech Republic	5	1	2	5	5
Denmark	5	2		2	1
Egypt, Arab Rep.	1				
Estonia	1				
Ethiopia	2				
Finland	4				
France	17	4	1	2	9
Germany	21	14	14	16	14
Ghana					1
Greece	1			5	
Hong Kong SAR, China	1			1	
Hungary				1	
Iceland	1				
India	2	3	2	3	4
Iran, Islamic Rep.	1				
Israel	2	3		5	5
Italy	2	2	2	9	5
Japan	4	4	2	4	4
Kazakhstan					4
Kenya	1				
Korea, Rep.	1	2			2
Lebanon	1			2	
Lithuania					1
Madagascar					2
Malaysia				1	1
Mexico	4	12	4	4	4
Netherlands	3	1	4		4
New Zealand	7	5	1	1	5
Nigeria	2	2	1		
Pakistan	1				
Panama	1	1		1	
Peru	2				
Philippines				1	1
Poland	3	3	4	2	6
Portugal	1			1	3
Qatar					1
Romania					2
Russian Federation	2	3			
Senegal					1
Singapore	1			1	
South Africa	4	18	12	11	8
Spain	11	5	8	15	13
Sweden	1	1	1		
Switzerland	4	8	6	7	11
Tanzania	1				
Thailand				1	1
Turkey					3
Uganda	2				
United Kingdom	60	27	35	31	39
Total count	317	201	154	220	228
% of registrants	18%	19%	12%	16%	17%

## Appendix 4 Profit and Loss Statement for Fiscal Years 2020–2023

**Table:**

Profit and loss by class					
January 2020 through December 2023					
		January—December 20	January—December 21	January—December 22	January—December 23
Ordinary Income/Expense					
Income					
40,050 · Advertising		0.00	0.00	0.00	0.00
40,100 · Donations		56,392.55	6075.00	5213.00	4568.00
40,150 · Donation—Amazon Smile		0.00	8000.00	0.00	0.00
40,200 · Grants					
40,210 · Elsevier Diversity Grant		0.00	0.00	0.00	0.00
40,220 · NSF IDEAS #1516939		0.00	0.00	0.00	0.00
40,221 · NSF Advancing Data (WI) #182688		0.00	0.00	0.00	0.00
40,230 · Wiley & Sons Grant in Aid		0.00	55,000.00	55,000.00	55,000.00
40,240 · Leakey Foundation Grant		0.00	0.00	30,000.00	0.00
Total 40,200 · Grants		0.00	55,000.00	85,000.00	55,000.00
40,300 · Interest Income		0.00	0.00	82.18	12,111.09
40,500 · Meeting Revenue					
40,000 · Auction		0.00	0.00	7954.50	8976.45
40,510 · Member Reduced Registration		680.00	1475.00	420.00	335.00
40,515 · Postdoc/Contingent Faculty Regi		30,715.00	18,880.00	21,480.00	23,452.00
40,520 · Member Registration		63,470.00	64,704.00	92,324.00	102,260.00
40,525 · Retired Member Registration		1040.00	0.00	0.00	920.00
40,530 · Non‐Member Reduced Registration		935.00	800.00	50.00	550.00
40,540 · Non‐Member Registration		20,020.00	5200.00	453.00	10,706.00
40,550 · Non‐Member Symposium Regist		11,760.00	4560.00	16,368.00	453.00
40,560 · Student—Member Registration		56,000.00	23,978.00	34,320.00	34,865.60
40,570 · Student—Reduced Registration		1685.00	775.00	786.00	700.00
40,580 · Student—Non‐Member Regist		19,565.00	10,695.00	13,178.00	16,130.00
40,583 · T‐Shirt Sales		0.00	1587.00	2904.00	4247.36
40,585 · Companion		0.00	0.00	0.00	0.00
40,592 · Tours		1729.00	0.00	210.00	0.00
40,595 · Sponsorship		350.00	8150.00	7000.00	4650.00
40,597 · Exhibitors		5000.00	1200.00	5275.00	14,250.00
40,599 · Room Credit		0.00	0.00	7488.41	22,773.93
40,500 · Meeting Revenue—Other		0.00	0.00	0.00	0.00
Total 40,500 · Meeting Revenue		212,949.00	142,004.00	210,210.91	245,269.34
40,600 · Membership					
40,610 · Regular		111,180.00	78,710.00	96,050.00	86,530.00
40,615 · Regular Developing Country		1280.00	920.00	840.00	600.00
40,620 · Regular Postdoc/Contingent Facu		21,840.00	13,545.00	16,695.00	14,595.00
40,630 · Special Member Postdoc/Continge		4830.00	3045.00	2940.00	2520.00
40,635 · Special Member Developing Count		0.00	0.00	40.00	40.00
40,640 · Special Member		1360.00	1190.00	1635.00	1020.00
40,650 · Life Member		0.00	400.00	0.00	0.00
40,660 · Student		53,755.00	28,665.00	37,830.00	36,530.00
40,670 · Student Developing Country		740.00	415.00	420.00	460.00
40,680 · Spouse Member		180.00	150.00	150.00	120.00
40,690 · Regular Retired		3070.00	2275.00	2145.00	1885.00
40,698 · Single Issue AJPA		30.00	0.00	0.00	0.00
40,699 · Print Journal		2090.00	0.00	0.00	0.00
40,600 · Membership—Other		0.00	0.00	0.00	0.00
Total 40,600 · Membership		200,355.00	129,315.00	158,745.00	144,300.00
40,800 · Royalties		141,520.00	0.00	0.00	0.00
49,900 · Miscellaneous Income		0.00	−1433.36	0.00	343.00
49,990 · Revenue—Suspense		0.00	0.00	0.00	0.00
50,010 · Meeting Refund/Cancellation		−149,486.00	−240.00	6013.08	−210.00
Total Income		**461,730.55**	**338,720.64**	**465,264.17**	**461,381.43**
Gross Profit		461,730.55	338,720.64	465,264.17	461,381.43
Expense					
60,000 · Annual Meeting Expenses					
60,004 · Abstracts & Programs		0.00	0.00	0.00	9853.19
60,006 · Attrition		0.00	0.00	0.00	0.00
60,008 · Audio & Visual		0.00	7440.00	61,261.32	50,799.09
60,010 · Auctions		0.00	0.00	242.76	330.00
60,015 · Childcare		533.95	0.00	8312.65	7980.95
60,020 · Coffee		0.00	0.00	29,994.30	24,380.34
60,024 · Committee Functions		0.00	0.00	1622.03	4469.55
60,028 · Contractor Services		0.00	0.00	0.00	0.00
60,030 · Exhibit/Spon Commission		3000.00	2500.00	0.00	2050.00
60,032 · Exhibits		1200.00	200.00	5125.00	6196.08
60,036 · Food Functions		0.00	0.00	11,466.05	10,095.74
60,040 · Hospitality		0.00	0.00	0.00	0.00
60,042 · Internet Meeting (Wifi)		0.00	0.00	5008.00	1224.00
60,044 · Invited Speakers		0.00	9500.00	3104.86	2372.65
60,046 · Local Arrangement Committee		0.00	0.00	3710.98	1110.42
60,047 · Meeting App		0.00	35,525.00	22,810.00	32,250.00
60,048 · Meeting Space		0.00	0.00	0.00	107.50
60,050 · Miscellaneous		750.00	0.00	7739.50	0.00
60,051 · NAVEX Code of Conduct Platform		2070.05	2000.00	0.00	3500.00
60,052 · Posters & Signs		0.00	0.00	7153.25	7270.00
60,056 · Program Committee		6677.90	290.69	6418.36	9850.10
60,059 · Reception—Closing		0.00	0.00	29,939.63	25,112.14
60,060 · Reception—General		0.00	0.00	56,919.38	74,687.41
60,064 · Reception—President		0.00	0.00	8123.63	10,388.66
60,068 · Registration		1950.00	2300.00	5782.50	1832.00
60,070 · Symposium Support		0.00	0.00	4747.36	12,179.86
60,072 · Student Expenses		0.00	0.00	0.00	228.65
60,074 · Supplies		0.00	0.00	0.00	414.87
60,076 · Teacher Workshop		0.00	0.00	0.00	0.00
60,078 · T‐Shirts		0.00	1558.98	2266.24	2672.68
60,080 · Tote Bags		0.00	0.00	2304.02	3192.16
60,084 · Volunteers		0.00	0.00	160.00	−88.00
60,000 · Annual Meeting Expenses—Other		0.00	0.00	0.00	0.00
Total 60,000 · Annual Meeting Expenses		16,181.90	61,314.67	284,211.82	304,460.04
60,100 · Awards					
60,110 · Pollitzer		3144.57	0.00	14,300.00	14,800.00
60,120 · Professional Development Awards		37,500.00	42,364.00	37,500.00	37,500.00
60,130 · Recognition Awards		192.95	0.00	713.82	2042.88
60,140 · Student Presentation Award		395.39	3250.00	5450.00	7300.00
60,100 · Awards—Other		0.00	464.00	132.53	578.17
Total 60,100 · Awards		41,232.91	46,078.00	58,096.35	62,221.05
60,200 · Bank Charges		797.85	0.00	0.00	0.00
60,210 · Merchant Fees		9278.77	9261.84	19,023.13	16,291.92
60,220 · Paypal Fees		1092.56	18.00	284.26	159.90
60,230 · Investment Fees		180.00	180.00	180.00	138.83
60,300 · Computer and Internet		154.63	198.97	523.78	808.20
60,310 · Web Hosting		2656.45	2600.00	2900.00	3100.00
60,320 · Web Master		6100.00	500.00	0.00	40,750.00
60,350 · Postage & Shipping		372.45	209.61	3006.45	5052.08
60,360 · Printing		56.88	0.00	4536.83	0.00
60,370 · Supplies		85.96	0.00	591.70	661.00
60,400 · Dues and Subscriptions		500.00	0.00	500.00	682.77
60,500 · Fees and Licenses		339.00	515.30	339.00	353.38
60,600 · Honoraria		200.00	0.00	0.00	0.00
60,700 · Insurance		10,726.82	7023.00	9472.18	9196.39
60,800 · Membership Servicing		96,999.96	96,999.96	96,999.96	96,999.96
60,900 · Officers & EC Expenses					
60,910 · Supplies		0.00	0.00	0.00	0.00
60,900 · Officers & EC Expenses—Other		0.00	0.00	0.00	0.00
Total 60,900 · Officers & EC Expenses		0.00	0.00	0.00	0.00
60,920 · Travel		0.00	736.65	9596.99	6620.84
60,925 · Travel—Secretariat		2869.51	0.00	5879.43	11,311.80
60,930 · VP Office		4000.00	12,000.00	8000.00	8000.00
61,010 · Accounting		47,186.00	48,099.50	43,204.75	36,152.25
61,020 · Legal		12,754.66	3300.00	890.00	1835.00
61,100 · Publisher Expense		148,014.55	0.00	0.00	0.00
63,000 · Grant Expense					
63,050 · NSF Grant					
63,051 · NSF—Participant Support Cost		0.00	0.00	0.00	0.00
63,055 · NSF—Other Costs		0.00	120.00	0.00	0.00
63,058 · NSF—Unallowable Costs		0.00	0.00	0.00	0.00
Total 63,050 · NSF Grant		0.00	120.00	0.00	0.00
63,100 · NSF IDEAS Grant Expenses					
63,105 · NSF IDEAS Participant Supp Cost		0.00	10,150.00	0.00	0.00
63,110 · NSF IDEAS Materials & Supplies		0.00	0.00	0.00	0.00
63,115 · NSF IDEAS Publication Cost		0.00	0.00	0.00	0.00
63,120 · NSF IDEAS Consultant Services		0.00	0.00	0.00	0.00
63,125 · NSF IDEAS Other Costs		320.51	0.00	0.00	0.00
63,135 · NSF IDEAS Matching Expenses		0.00	0.00	0.00	0.00
63,140 · NSF IDEAS Unallowable Costs		0.00	0.00	0.00	0.00
Total 63,100 · NSF IDEAS Grant Expenses		320.51	10,150.00	0.00	0.00
63,200 · Wiley Grant in Aid		0.00	0.00	0.00	0.00
63,300 · Elsevier Grant		0.00	500.00	2907.22	0.00
Total 63,000 · Grant Expense		320.51	10,770.00	2907.22	0.00
67,000 · Contribution/Organization Supp		7500.00	0.00	0.00	0.00
69,800 · Uncategorized Expenses		0.00	0.00	0.00	0.00
69,850 · Miscellaneous Expense		114.24	0.00	0.00	1387.50
79,000 · Overhead and Indirect Costs		0.00	360.00	360.00	360.00
Total Expense		**409,715.61**	**300,165.50**	**551,503.85**	**606,542.91**
Net Ordinary Income		**52,014.94**	**38,555.14**	**−86,239.68**	**−145,161.48**

## Appendix 5 Member Applications

### New Regular Postdoc/Contingent Members (31)

Carmen Alonso‐Llamazares

Julia Aramendi

Mikel Arlegi

Madeline Atwell

Lucie Biehler‐Gomez

Shannon Black

Kathryn Driscoll

Wren Edwards

Ulises Espinoza

Marc Gaede

Guido Alberto Gnecchi Ruscone

Hester Hanegraef

Malene Friis Hansen

Maureen Hickey

Paul Hooper

Ellen Kendall

Ross Kendall

Salvatore Liccardo

Jordan Martin

Alexandria Orozco

Thais Pansani

Rebeka Rmoutilova

Kaleb Sellers

Anne Marie Sohler‐Snoddy

Maria Spyrou

Cathryn Townsend

Alessandra Varalli

Elizabeth Veatch

Miguel Vilar

Franziska Wagner

Deming Yang

### New (and Previously Expired) Regular Members (51)

Bridget Alex

Clark Barrett

Anna Behrensmeyer

Šárka Bejdová

Amy Boddy

Matthew Borths

Elissa Bullion

Noel Cameron

Liran Carmel

Isaura Cruz

LaKisha David

Michelle de Saint Pierre

Dorothy Dechant

Kimberly Dingess

Leyla Djansugurova

Peterson Faina

Rafał Fetner

Bonny Ford

Michael Frachetti

Rebecca Gibson

Christopher Heesy

Taylor Hermes

Tobias Houlton

John Lindo

Chin‐hsin Liu

Elizabeth Lonsdorf

Joseph Lubega

Jessica Lynch

Francesco Marino

Nancy Minugh‐Purvis

Michael Muehlenbein

Papa Ibnou Ndiaye

Pablo Nepomnaschy

Alex Piel

Dario Piombino‐Mascali

Cosimo Posth

Niraj Rai

Jonah Ratsimbazafy

Luis Ríos

Hélène Rougier

John Rowan

Nohemi Sala

Rebecca Sear

Amy Shapiro

Kathryn Smith

Jeff Stibel

Martin Surbeck

Shintaro Suzuki

Alicia Ventresca‐Miller

Richard Wrangham

Qun Zhang

### New Special Members (16)

Samantha Beck

Kristina Cockerille

Eleanor Crookes

Sarah Fachnie

Cindy Gill

Deepali Haldar

Meghan Khalsa

Martin Levin

Roxanne Mayoral

Elizabeth Meyer

Mary Pyles

Margarita Reyes

Nicole Saenz

Jeff Stibel

Marshall Ustrnul

Margarita (Maggie) Villarreal

### New Student Members (276)

Elyse Adams

Florence Aghomo

Elaine Aguayo Ortiz

Larry Aguirre

Bhavna Ahlawat

Isabella Almeida de Oliveira

Youbin An

Tyler Andres‐Bray

Lillian Antonelli

Kaylee Appleton

Caitlin Archdeacon

Olivia Ares

Ayken Askapuli

Ruth Aul

Abilene Ayala

Alleyia Bailey

Niguss Baraki

Skye Barger

Matthew Barker

Keegan Beane

Nikki Bennett

Daniel Biggs

Karis Bittner

Leah Blair

Ashley Blake

Grace Bocko

Jazmin Borrayo

Erin Boyle

Claire Brandes

Clara Brandon

Isabella Bristol

Brittany Brown

Keri Burge

Caroline Burks

Madison Butler

Austin Butler

Ileana Buzic

Beatrice Caffe

Ansley Cahill

Stevie Capps

Jessica Castaño

Vivian Cheng

Catherine Clayton

Peyton Cleaver

Courtney Clough

Maxwell Cockerill

Valerie Contreras

Amanda Cooke

Mariha Corbin

Quentin Cosnefroy

Christina Costa

Isabella Cowan

Camille Cronkhite

Mackenzie Cross

Evan Cunningham

Connor Davis

Laurel Davis

Edwin de Jager

Mary Dewitt

Brianna Dittmer

Kaelyn Dobson

Anastasia Dorenbusch

Rhianna Drummond‐Clarke

Dejing Du

Sarah Durham

Aparna Dwivedi

Morgan Elmore

Aiden Eylward

Leah Fehringer

Susan Femino‐Floyd

Elise Ferguson

Henrike Fiedler

Emily Fields

Natalie Finnegan

Veronica Flores‐Guillen

Anika Florin

Jordan Franklin‐Garza

Caitlin Frie

Tania Galvez

Tessa Garces

Cassandra Garcia

Lucy Garrison

Hannah Giberti

Haechan Gill

Olivia Golubowski

Eimy Gonzalez Alvarez

Owen Goodchild

Ashe Gooden

Miranda Goodstein

Madison Grant

Tanyaradzwa Guduza

Sara Gutierrez

Matthias Hackl

Julia Haffie

Rachael Hall

Meutia Hanafiah

Halle Hangen

K.T. Hanson

Abigail Harward

Margaret Hassler

Kale Hawks

Sarah Heath

Lucy Henderson

Maiya Higa‐Okohira

Mason Hintermeister

Kelly Hoag

Tara Hobbs

Talia Hoffman

Rainie Hoogendoorn

Laura Howard

Magdalena Humphrey

Noah Hunt

Sydney Hunter

Delphi Huskey

Nadine Hussein

Skye Jackson‐Sims

Robin James

Maegan Jankowski

Jyhreh Johnson

Amanda Jokela

Gabrielle Jones

Mason Jones

Katelyn Kallman

Shanna Kang

Victoria Kashkin

Chloe Keefe

Janine Rose Klein

Sophie Klitgaard

Paul Klostermann

Shuxin Kong

Keegan Krause

Greta Kuhne

Adrienne Kuziek

Thomas LaBarge

Dawson Lamb

Jennavieve Lambeth

Anthony Lanfranchi

Meghan Langlois

Francis Lawrence

Lydia Lehman

Malefeu Lethuba

Ming Fei Li

Peishu Li

Alessandro Lisi

Junjian Liu

Yan Liu

Brianna Liu

Ruizhe Liu

Halee Lott

Morgann Lucas

Autumn Lucas

Amanda Magpiong

Kira Marchek

Jessica Martin

Natalie Marud

Jack McBride

Alina McCloud

Emma McCollister

Alexandra McDougle

Bridget McGann

Kelly McGehee

Alyssa McGrath

Zoen McLachlan

Peter Mercier

Molly Militello

Emily Miller

Taylor Mitchell

Matthew Morley

Angela Mossor

Marineh Mousalu

Monica Msechu

Mackenzie Murray

Lavania Nagendran

Anamika Nanda

Eliette Noromalala

Tomasz Nowak

Emily Opack

Michael Orellana

Diego Ortiz

Julianne Paige

Maria Jose Palma

Kevin Palmisano

Vaidik Pandya

Ana Pantoja‐Pérez

Cristiano Parmeggiani

Felipe Parodi

Melissa Patton

Lucille Pedersen

Reina Perez Munoz

Marcela Pfaff Nash

Shelby Pirtle

Lia Plankenhorn‐Farrell

Elizabeth Poplavska

Melina Porro

Amanda Pregibon

Luke Quarles

Aditi Raghunath

Joel Ramirez

Gemma Ranson

Levi Raskin

Harimanjaka Rasolonjatovo

Kenna Reed

Mercedes Rego

Rebecca Reid

Ana Reif

Lucia Rejzek

Nicholas Reul

Madeja Rheddick

Drake Rinks

Rachel Robrecht

Madison Rodriguez

Juan E. Rodriguez Rodriguez

Isabel Roji

Jennifer Rollins

Domenic Romanello

Olivia Romero

Emilia Rose

Aigerim Rymbekova

Emma Sak

Anna Samsonov

Liya Samuel

Daniella Sandoval

Shivani Sanger

Jordyn Sauser

Kendra Scheer

Allyson Schmidt

Emily Schopmeyer

Jenna Mae Schultz

Cecilia Sena

Min Woo Seo

Taylor Seupaul

Jordan Shaffer

Samantha Shaw

Catherine Shipman

Alana Spearman

Arielle Sperber

Hannah Stottlemyre

Rebekah Stowe

Josie Taylor

James Tetreau

Zoe Thomas

Chelsea Thompson

Edward Thum

Emma Tinker‐Fortel

Mia Tomeo

Cassidy Tomlinson

Olivia Traweek

Amariah Trevino

Lillie Turpin

Alexandra Tutwiler

Samantha Unwin

Jose Urban Aragon

Sebastian Vagner

Mirina Van Der Veen

Marissa Vasquez

Steffi Vassallo

Abigail Vaughn

Yarinid Velez Hernandez

Ariel Vieira

Sahara Vilchis

Marissa Vink

Desiree Wagner

Xue Wang

Keane Wanza

Elizabeth Ware

Monica Warner

Kelly Watson

Sofia Weaver

Noelle Wilcox

Carly Wisner

Joseph Won

Alice Yan

Chloe Yingling

Emma Young

Jeremy Yuen

Andreanna Zaverl

### Student Member to Regular Post‐Doc/Contingent Member (33)

Amy Anderson

Jacob Aronoff

Christina Balentine

Andrea Blackburn

Kelly Blevins

Alyson Caine

Kristine Joy Chua

Joanna Ciesielska

Miranda Cosman

Dany Coutinho Nogueira

Jacob Feder

Stephanie Fox

Allison Ham

Alexandra Kralick

Austin Lawrence

Ignacio Lazagabaster

Victoria Lockwood

Cait McPherson

Sophia Mills

Hannah Moots

Jonathan Paige

Evelyn Pain

Taylor Paskoff

Megan Petersdorf

Kristen Rayfield

Ashly Romero

Caitlin Smith

Christopher Smith

Catherine Taylor

Sheila Nicole Torres Tamayo

Shevan Wilkin

J. Suzi Wilson

Katrina Yezzi‐Woodley

### Student Member to Regular Member (20)

Wendy Black

Kristen Broehl

Ryan Campbell

Stephanie Canington

Elaine Chu

Andreana Cunningham

Aja Lans

Carrie Miller

Alexis O'Donnell

Tiffany Pan

Katherine Reinberger

Kaitlyn Sanders

Keegan Selig

Chalachew Seyoum

Maximilian Spies

Maxx Toler

Trent Trombley

Devin Ward

Christopher Wolfe

Shiyu Yang

### Student Member to Special Member (9)

Ashley Baeza

Megan Carey

Amy Chan

Bronte Cunningham

Eric Frauenhofer

Christopher Holenbeck

Olivia Mehalko

Maxine Neiberg

Diego Stokes‐Malave

### Special Member to Student Member (4)

Aparna Chandrashekar

Emilee Hart

Abigail Musch

Eric Schissler

### Special Member to Regular Member (2)

Eric Anderson

Christine Bail

## Appendix 6 Pollitzer Award Winners

**Table:**

Chelsea Rose	University of Central Florida
Madison Grant	CUNY
Elizabeth Poplavaska	Boston University
Rancis Short	Boston University
Megan Langlois	McMaster University
Lydia Bailey	Mississippi State University
Madeline Kelly	University of Chicago
Jessica Martin	Boston University
Julie Strain	CUNY
Abigail Houkes	University of Illinois Champagne Urbana
Keegan Krause	Northwestern
Rebecca Reid	University of Dundee
Ayken Askapuli	University Of Wisconsin Madison
Rebecca Cook	Duke
Madison Bradley‐Cronkwright	Duke
Amanda Cooke	McMaster University
Margaret Furtner	Louisiana State University
Matthias Hackl	University of Calgary
Allyson Simon	The Ohio State University
Anna Samsonov	Baylor
Luke Quarles	The University of Buffalo
Madison Butler	University of New Mexico
Rebecca Seegraber	University of Utah
Laura Pott	University of New Mexico
Clara Brandon	University of Michigan
James Tetreau	University of Calgary
Alexandra McDougle	Columbia
Livia Santos	George Mason University
Mary DeWitt	Purdue
Nadine Hussein	University of Calgary
Isabel Roji	San Diego State University

